# Beneficial effects of foreign language learning and aerobic exercise on dentate gyrus volume and mnemonic discrimination in healthy older adults: Results from a randomized controlled trial

**DOI:** 10.1162/IMAG.a.1306

**Published:** 2026-07-24

**Authors:** Sarah E. Polk, Yana Fandakova, David Berron, Sandra Düzel, Andreas M. Brandmaier, Simone Kühn, Ulman Lindenberger, Elisabeth Wenger

**Affiliations:** Center for Lifespan Psychology, Max Planck Institute for Human Development, Berlin, Germany; Clinical Cognitive Neuroscience, DZNE German Center for Neurodegenerative Diseases, Magdeburg, Germany; Department of Psychology, University of Trier, Trier, Germany; Friede Springer Cardiovascular Prevention Center, Charité – Universitätsmedizin Berlin, Berlin, Germany; Department of Psychology, MSB Medical School Berlin, Berlin, Germany; Max Planck UCL Centre for Computational Psychiatry and Ageing Research, Berlin, Germany; Lise Meitner Group for Environmental Neuroscience, Max Planck Institute for Human Development, Berlin, Germany; Department of Psychiatry and Psychotherapy, University Clinic Hamburg-Eppendorf, Hamburg, Germany; Institute for Mind, Brain and Behavior, HMU Health and Medical University, Potsdam, Germany

**Keywords:** healthy aging, aerobic exercise, language learning, hippocampal subfields, mnemonic discrimination

## Abstract

Protecting hippocampal structures, such as the *cornu ammonis* 3 and dentate gyrus (CA3/DG), that are tied to mnemonic discrimination could be a promising approach to mitigate the age-related decline in episodic memory. This study examines the potential benefits of cognitive and physical engagement, specifically language learning and moderate aerobic exercise, for maintaining hippocampal subfield integrity and memory performance. In a randomized controlled trial involving 142 healthy older adults (aged 63–78 years), we investigated the effects of a 6-month regime comprising language learning, aerobic exercise, and their combination. Participants were assigned to one of four groups: an active control, language-only, exercise-only, or combined language and exercise group. CA3/DG volume remained stable in the language-only and combined groups, while the non-language groups showed a significantly greater decrease in volume, suggesting that foreign language learning may protect against normal aging-related CA3/DG volume loss. Comparatively, the exercise-only and combined groups showed significant improvements in mnemonic discrimination performance, while the non-exercise groups did not, indicating that aerobic exercise can improve this aspect of memory in older adults. Notably, the combined group was the only group to exhibit both preserved CA3/DG volume and improved mnemonic discrimination. No evidence for a boosting effect of the combined intervention on either outcome was observed. This suggests that, while the effects of language learning and aerobic exercise may be independent at the level of measured outcomes, they provide complementary benefits when combined. Finally, the initial volume of the subiculum was found to predict changes in mnemonic discrimination performance across all groups, indicating that this region may play a potential role in episodic memory changes over time. Contrary to our expectations, changes in hippocampal subfields were not directly associated with changes in mnemonic discrimination. In conclusion, this study highlights the potentially separate roles of foreign language learning and aerobic exercise in enhancing aspects of episodic memory in older adults; future research may address whether their combination may yield broader functional benefits than either intervention alone.

## 1 Introduction

Episodic memory function, which typically begins to deteriorate in healthy older adults around the age of 50–60 years, is underpinned by mnemonic discrimination (Nyberg et al., 2012; Rönnlund et al., 2005; Schaie, 1994). This ability to discern between similar but distinct stimuli tends to worsen with age (Berron et al., 2018; Burke et al., 2010; Fandakova et al., 2018; Stark et al., 2010; Toner et al., 2009). Older adults often exhibit an increased tendency towards false alarms, incorrectly identifying new items as familiar. This reduced precision may significantly contribute to the episodic memory deficits observed in this age group (Stark et al., 2013; Yeung et al., 2013).

Numerous functional magnetic resonance imaging (MRI) studies have highlighted the critical role of hippocampal subfields, particularly the *cornu ammonis* 3 (CA3) and the dentate gyrus (DG), in pattern separation, which is the neural process that underlies mnemonic discrimination (Bakker et al., 2008; Berron et al., 2016; Lacy et al., 2011; Yassa et al., 2010). Furthermore, cross-sectional research investigating the structural integrity of the CA3/DG has revealed a positive correlation between the volume of this region and performance on mnemonic discrimination tasks (Bennett et al., 2019; Kern et al., 2021; Shing et al., 2011).

Hippocampal subfield volume is known to decrease with age (e.g., Baumeister et al., 2025; Daugherty et al., 2016; de Flores et al., 2015; Zhang et al., 2020). If this loss contributes longitudinally to less precise pattern separation in older adults, those experiencing more substantial volume loss in the CA3/DG should theoretically exhibit worse mnemonic discrimination performance compared to their peers of the same age who do not suffer the same degree of volume loss in these subfields (Cabeza et al., 2018; Lindenberger, 2014; Nyberg & Lindenberger, 2020; Nyberg & Pudas, 2019; Nyberg et al., 2012).

The hippocampus, while particularly susceptible to aging-related atrophy, is also impressively receptive to positive influences such as experience-induced plasticity (Walhovd et al., 2016). This potential for change has made the hippocampus a prime target for interventions that aim to decelerate, or even reverse, age-related deterioration in its structural integrity, thereby slowing the decline of episodic memory in older age (Thomas & Gutchess, 2020). Research into lifestyle factors has pointed toward certain activities, notably cognitive enrichment and physical activity, as potential safeguards against age-related hippocampal deterioration in older adults (Lindenberger, 2014; Nyberg & Lindenberger, 2020; Nyberg et al., 2012). These protective measures represent promising avenues for fostering brain health and cognitive resilience in the aging population.

Regarding cognitive enrichment, recent interest for foreign language acquisition has surged, given its potential implications for hippocampal plasticity (Antoniou & Wright, 2017; Li et al., 2014). However, investigations into the effects of foreign language learning in aging samples remain sparse (see Ware et al., 2021 for a review). While a few studies involving young adults have reported structural changes in language-related brain regions and the hippocampus, no impact on mnemonic discrimination performance has been observed (Bellander et al., 2016; Mårtensson et al., 2012). To our knowledge, the only study to date hypothesizing a similar effect of foreign language learning on the hippocampus in older adults yielded inconclusive results (Nilsson et al., 2021). In this intervention, 65- to 75-year-old native Swedish speakers were assigned to either a group that learned Italian for 11 weeks or a relaxation group (Berggren et al., 2020; Nilsson et al., 2021). No evidence was found for language learning’s effect on hippocampal structural integrity (Nilsson et al., 2021), nor for its impact on various cognitive domains, including associative memory (Berggren et al., 2020). Notably, no tasks specifically testing mnemonic discrimination were administered. Therefore, the current evidence is inconclusive as to whether language learning can induce changes in hippocampal volume in older adults that could preserve mnemonic discrimination. Moreover, no studies have yet explored the impact of foreign language learning on the structural integrity of specific hippocampal subfields.

Regarding physical activity, aerobic exercise has gained significant attention, with studies examining its effects on hippocampal volume and episodic memory in aging individuals (Düzel et al., 2016). Research has reported increases in hippocampal volume following 6 months to a year of exercise in older adults (Erickson et al., 2011; Maass et al., 2015), as well as volume increases specific to the DG in young and middle-aged adults (Frodl et al., 2020). These changes were associated with improvements in spatial memory performance (Erickson et al., 2011) and tasks requiring mnemonic discrimination between visually similar shapes (Frodl et al., 2020; Maass et al., 2015). Taken together, aerobic exercise appears to be a promising intervention to slow age-related hippocampal atrophy and enhance episodic memory performance in older age (see Aghjayan et al., 2022 for a systematic review). Nevertheless, the specific association between exercise-induced changes in subfield volume and mnemonic discrimination in healthy older adults remains unexplored.

Finally, it has been postulated that cognitive and physical training might interact, resulting in enhanced outcomes compared to single-type training (Bamidis et al., 2014; Hötting & Röder, 2013; Kempermann et al., 2010; Kraft, 2012; Lustig et al., 2009). Mechanistically, physical exercise might trigger potential cellular changes, like neurogenesis (Biedermann et al., 2016) and synaptogenesis, while cognitive stimulation could steer this potential towards meaningful structural changes (Fissler et al., 2013). Supporting this hypothesis, several intervention studies have reported beneficial outcomes from combined cognitive and physical exercise intervention in older adults. These include significant improvements in a composite memory score (Fabre et al., 2002), a cognitive function composite (Oswald et al., 2006), and more recently, a mnemonic discrimination task (Cui et al., 2023). Importantly, these improvements were the most pronounced in combined training groups compared to single training-type groups. Collectively, these findings provide some preliminary evidence that a combined cognitive and physical training regimen may be advantageous for preserving hippocampal integrity in aging humans, leading to gains in, or maintenance of, mnemonic discrimination compared to either cognitive or physical training alone. However, the combined effects of foreign language acquisition and aerobic exercise remain unexplored, marking an intriguing area for future research.

In the current study, we explored the effects of 6 months of cognitive and physical training, both individually and combined, on hippocampal subfield volume and a mnemonic discrimination task in older adults. For cognitive training, we implemented a foreign language acquisition intervention, facilitated through a digital application on a tablet 6 days a week, supplemented with a weekly language lesson. The physical training was delivered in the form of a personalized at-home interval training program, accessed via a tablet and paired with a bicycle ergometer. While previous cognitive and exercise interventions have primarily examined total hippocampal volume using lower-resolution imaging techniques, we focused on specific hippocampal subfields in older adults. To this end, we utilized high-resolution structural hippocampal MRI and automated subfield segmentation of the hippocampal body (Bender et al., 2018) to explore the effects of language training and aerobic exercise on specific hippocampal subfields.

We hypothesized that regular cognitive and physical activity would individually slow the age-related loss of hippocampal volume, and that combined training might possibly amplify these effects. We also anticipated training-related improvements in a mnemonic discrimination memory task associated with both language learning and physical exercise, and that combining language learning and exercise training could potentially offer enhanced benefits beyond either training alone. Finally, we expected an association between changes in hippocampal subfield volume and mnemonic discrimination. Specifically, the attenuation of volume loss should correspond to the maintenance or improvement of performance on the mnemonic discrimination task.

## 2 Methods

### 2.1 Sample and study design

Healthy, sedentary older adults (63–78 years old) participated in the AKTIV: Aktives Altern für Körper und Geist [active aging for body and mind] study, which was conducted at the Max Planck Institute for Human Development, Berlin (MPIB) as part of the Energizing the Hippocampus in Aging Individuals (EnergI) Consortium. The full study protocol can be found in Wenger et al. (2022) and the Consolidated Standards of Reporting Trials (CONSORT) 2010 Checklist (Schulz et al., 2010) can be found in the Supplementary Materials (Supplementary Table S1); here, we report relevant details for the current analyses. The study was approved by the ethics committee of the German Society for Psychology (DGPs) and informed consent was collected from all participants.

Volunteers were recruited through newspaper advertisements and previous participation in other studies,^1^ and were invited to participate if they had no MRI contraindications, were able to meet the time requirements of the study (ca. 45 minutes a day for 6 months), were right-handed, had no history of head injury or a medical, neurological, or psychological disorder, and were not taking any medication affecting memory function. Individuals who could speak a Romance language or were proficient in more than one language in addition to German, as well as those who engaged in aerobic exercise more than once every 2 weeks were not eligible for participation.

Qualifying volunteers (*N* = 201) were sequentially pseudo-randomly assigned to one of four training groups: (1) an active control group (ACG; *n* = 48), (2) a language group (LG; *n* = 48), (3) an exercise group (EG; *n* = 52), and (4) a combined language and exercise group (L+EG; *n* = 53). The first individual to be invited was assigned to the ACG, the second to the LG, the third to the EG, the fourth to the L+EG, and so on. Couples and friends who were invited to participate were assigned to the same intervention group, as participants were blinded to the existence of other groups. Participants were then invited to a baseline comprehensive physical examination by a sports medicine physician, where 22 individuals were excluded due to pre-existing medical conditions. All remaining participants underwent baseline testing including an MRI session and a comprehensive cognitive battery before beginning their assigned training (T1). Before the respective training regimes started, 20 further participants dropped out citing disinterest or claustrophobia. MRI scans and cognitive tests were repeated after 3 months of training (T2), and again after 6 months (T3), when participants also underwent a final physical assessment. Over the course of the study, 17 participants dropped out, leaving a final participant count of *N* = 142.

**Fig. 1.**
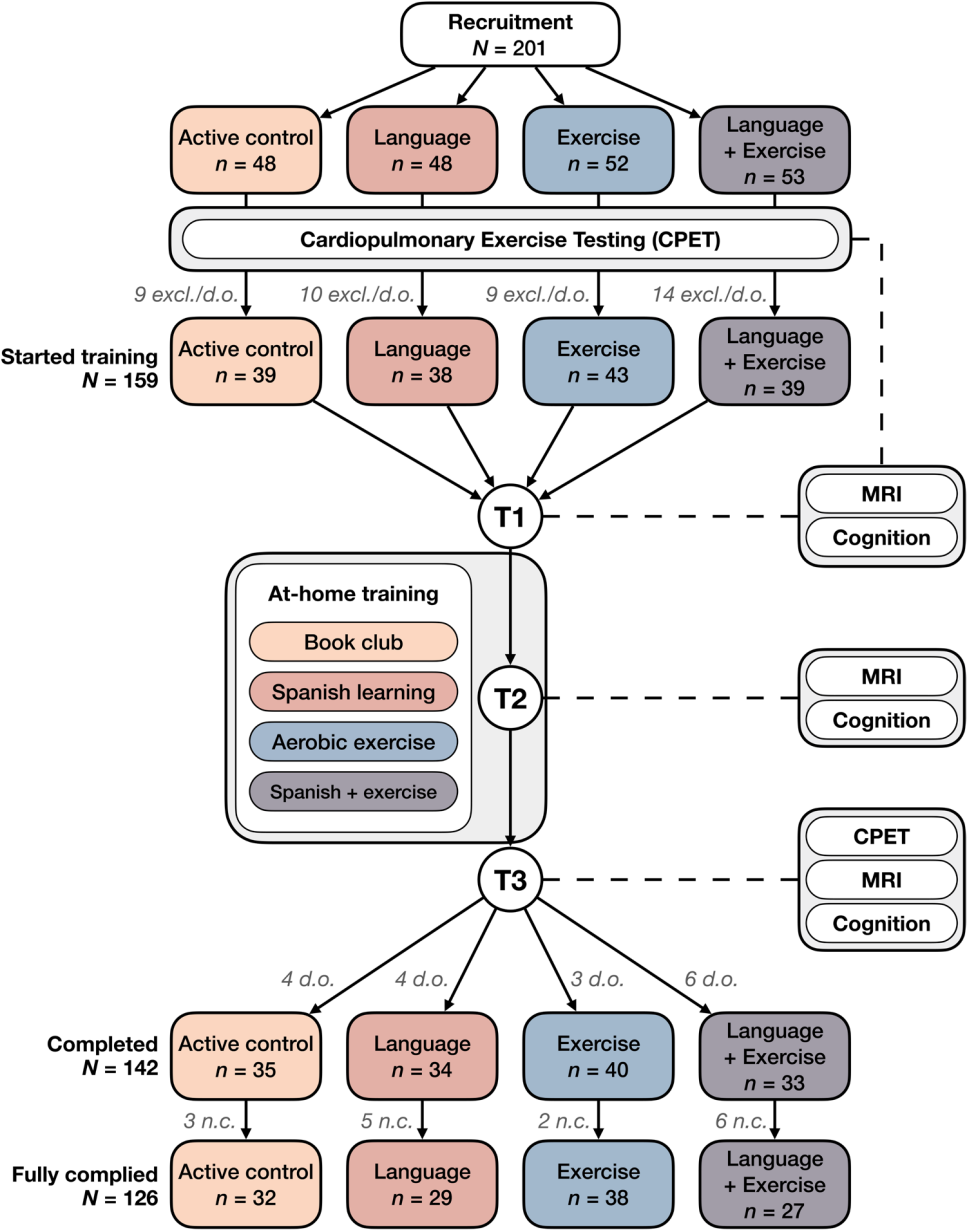
Study design with number of participants per group, including exclusions, drop-outs, and non-compliant participants per group. Excl. = excluded participants, d.o. = drop-outs, MRI = magnetic resonance imaging, T1 = time point 1 (baseline), T2 = time point 2 (3 months), T3 = time point 3 (6 months), n.c. = non-compliant participants. CPET at T1 was used to determine whether participants met certain health-based exclusion criteria. Adapted from Wenger et al. (2022) with permission.

Compliance to the interventions was defined as completing at least 1,890 minutes (90 minutes × 21 weeks) of study-related activity during the study; in total, 15 individuals did not reach this threshold. One additional participant experienced pervasive technical issues with the ergometer for the duration of the study and is considered non-compliant in the current analyses. In addition to the full data from compliant participants (*n* = 126), data from the baseline measurements, but not from T2 or T3, from those participants who were considered non-compliant (*n* = 16) were included in the current analyses, as compliance was unrelated to variables of interest before the intervention (see Supplementary Table S2), but could have affected the rate or magnitude of change in certain outcomes. See Figure 1 for a timeline of the study as well as group-wise drop-out and non-compliance rates. In total, *n* = 35 participants completed the study in the ACG (fully complied: *n* = 32), *n* = 34 in the LG (fully complied: *n* = 29), *n* = 40 in the LG (fully complied: *n* = 38), and *n* = 33 in the L+EG (fully complied: *n* = 27).

### 2.2 Interventions

**Fig. 2.**
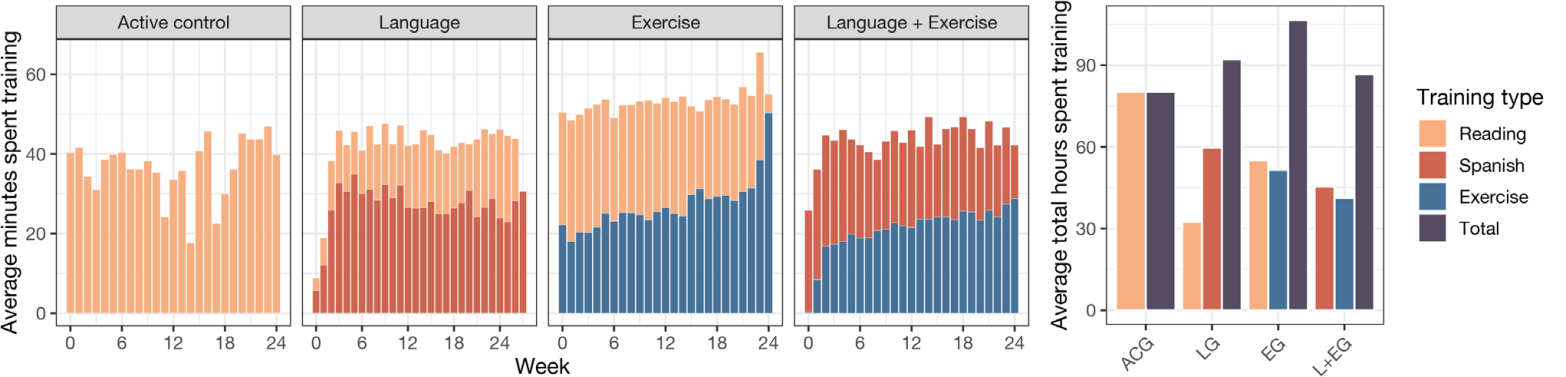
Time spent training (reading, learning Spanish, and/or exercising) each week, averaged across participants, and total time spent training during the study, averaged across participants. See Supplementary Table S3 for statistics regarding time spent training.

The interventions are briefly discussed in the following; further details can be found in Wenger et al. (2022). The interventions were designed to be completed largely unsupervised in participants’ own homes, with weekly in-person group sessions. In this way, we investigated the effects of lifestyle interventions that may be more accessible to older adults than the typical in-lab training completed under supervision. Participants in each intervention group aimed to engage in study-related activities at home for 45 minutes a day, 6 days a week, at any time of the day (see Figure 2 for a summary of time spent training). They attended weekly hour-long group sessions in person, where they engaged in a relevant activity with other participants (5–10 participants per session) in their respective groups; this was done in all groups so that participants would have equivalent social interaction related to participation in the study.

Participants in the ACG read pre-selected literature in German on a tablet (Lenovo TB2-X30L TAB) for ca. 45 minutes daily for at least 6 days per week. At the weekly ACG sessions, participants discussed short literary excerpts mediated by external facilitators.^2^

Participants in the LG used the Babbel application (Babbel, Lesson Nine GmbH) on a tablet to learn Spanish for ca. 30 minutes a day, or until they completed one lesson, 6 days a week. These lessons included learning vocabulary (words and phrases) as well as grammatical syntax. LG participants also read pre-selected literature on the tablet for an additional 15 minutes a day. For the weekly LG sessions, participants attended a Spanish class led by an external instructor.

Participants in the EG engaged in moderate aerobic exercise three to four times a week using a bicycle ergometer (DKN Ergometer AM-50) and an interval training application with personalized settings (e.g., starting resistance, interval length; determined by the attending physician at the first physical assessment) programmed onto a tablet. EG participants also read on the tablet for an additional 15 minutes on days when they trained or for 45 minutes when they did not. At the weekly EG sessions, participants engaged in a toning and stretching class led by an external instructor.

Participants in the combined L+EG learned Spanish 6 days a week and engaged in aerobic exercise 3–4 days a week, as described above. For the weekly L+EG sessions, participants attended a Spanish class, led by an external instructor.

### 2.3 Baseline measures, primary outcome measures, and preprocessing

For the sake of brevity, full details regarding the in-person measurements can be found in the Supplementary Methods, and relevant points are summarized in the following.

Physical fitness was quantified as peak oxygen uptake (VO_2_peak) using spirometry while the participant pedaled on a bicycle ergometer to subjective exhaustion (Bosquet et al., 2002; Braumann et al., 2004) and relativized by body weight in kilograms (Dickhuth & Badtke, 2010), which is a standard measurement of endurance performance (Bassett & Howley, 2000).

The Digit Symbol Substitution Task (DSST; Wechsler, 1981) was used as a general measure of cognitive health (Jaeger, 2018) to ensure that all groups had comparable cognition at baseline.

Brain images were acquired using a 3T Magnetom Trio MRI scanner system (Siemens Medical Systems, Erlangen, DE; software version VB17a) using a 32-channel radiofrequency (RF) head coil. A T1-weighted 3D magnetization-prepared rapid gradient echo (MPRAGE) sequence and a high-resolution, T2-weighted 2D turbo spin echo (TSE) sequence localized on the hippocampus were acquired at all three time points. See Supplementary Methods for sequence parameters.

**Fig. 3.**
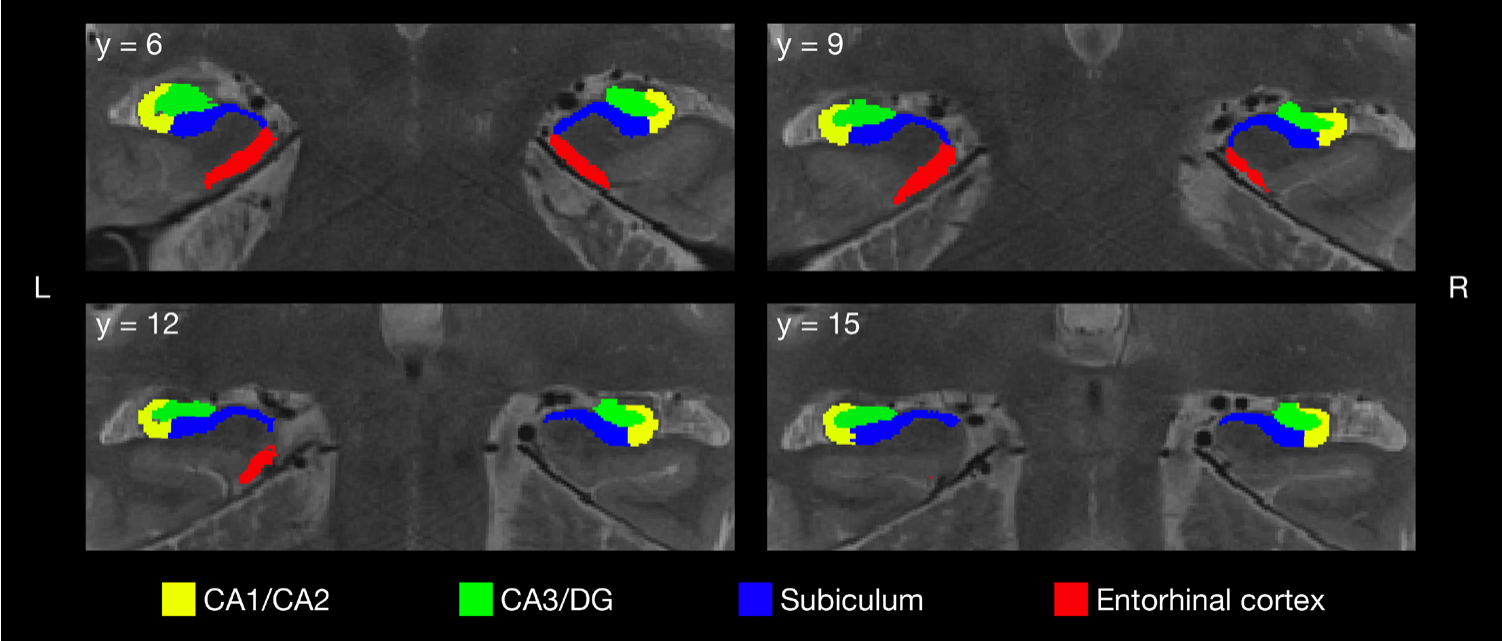
Example of results of the hippocampal subfield segmentation performed with ASHS seen on various coronal slices. CA = cornu ammonis, DG = dentate gyrus, L = left, R = right.

To extract hippocampal subfield volumes, the anterior and posterior limits of the bilateral hippocampal bodies were first manually demarcated by two raters who were blinded to participant ID, training group, and measurement time point (see Supplementary Methods for more details regarding the range). ASHS segmentation was then applied using a customized atlas that has previously been validated for use in healthy older adult samples (Bender et al., 2018). To correct for segmentation errors at the anterior and posterior limits of the hippocampal body, data were truncated to only those slices within the manually defined ranges to capture only the hippocampal body. Right and left CA1/CA2, CA3/DG, subiculum, and ERC volumes in mm^3^ were then extracted (see Fig 3). Finally, volumes of each subfield were adjusted for intracranial volume (ICV, calculated using the *Estimate TIV and global tissue volumes* module in CAT12) using an analysis of covariance approach (Raz et al., 2005): adjusted volume = raw volume − *b* × (ICV − mean ICV), where *b* is the slope of regression of subfield volume on ICV. Adjusted subfield volumes were used in all subsequently described analyses.

**Fig. 4.**
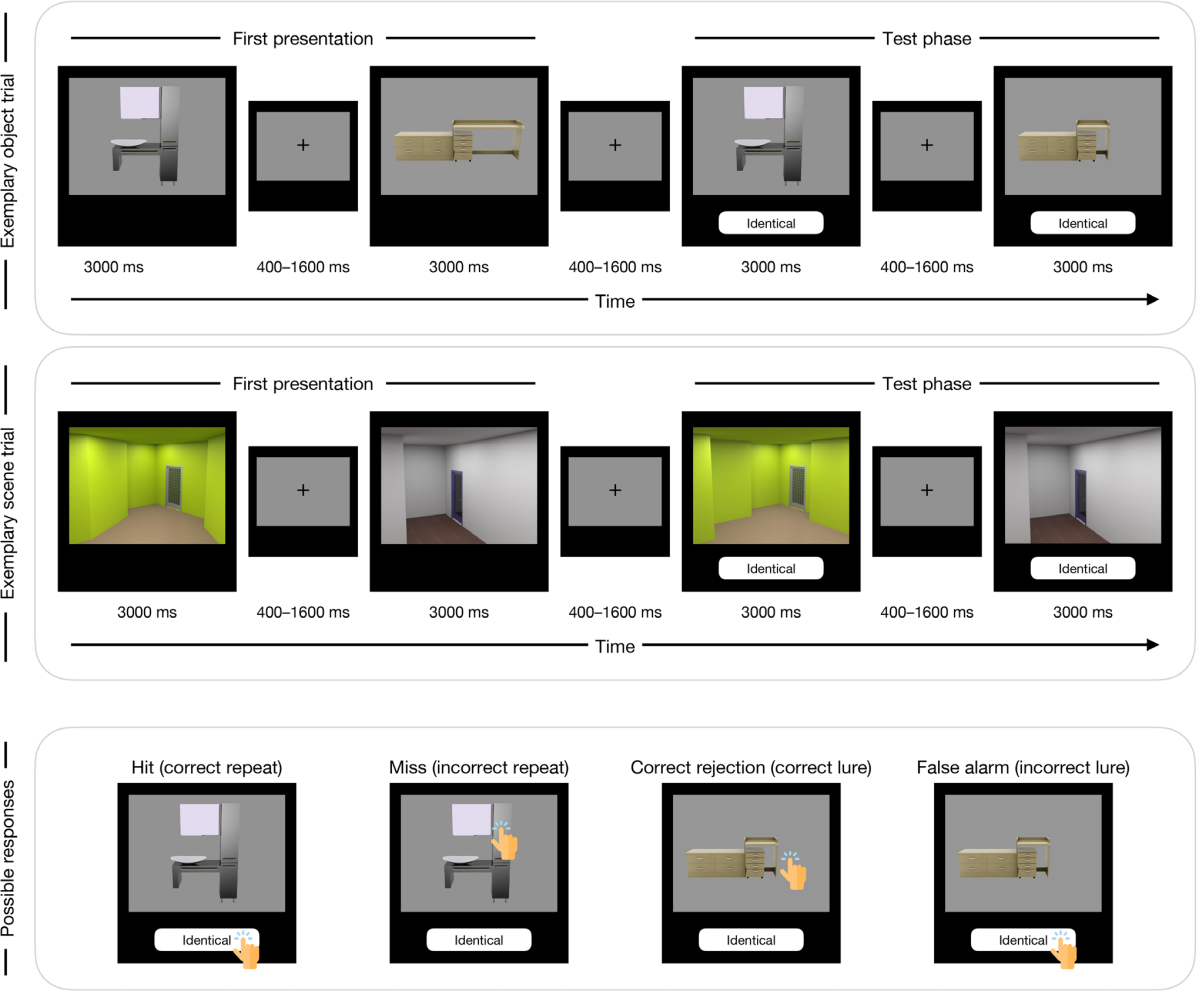
Examples of an object and a scene trial in the Mnemonic Discrimination Task for Objects and Scenes. Figure adapted from Berron et al. (2018) with permission.

To assess mnemonic discrimination, the Mnemonic Discrimination Task for Objects and Scenes (MDT-OS; adapted from Berron et al., 2018) was administered at all three time points on a touchscreen computer with a stylus. Each trial consisted of two images (first presentation) plus a repeat or lure version of each (test). Participants indicated if they thought each test image was a repeat by pressing a button labeled “Identical” with the stylus or a lure by indicating where they thought the image had changed (see Fig. 4 for an example of a single trial and answer options). See Supplementary Methods for more details regarding the stimuli and administration.

To ensure data quality, responses were discarded if reaction times were less than 200 ms (812 responses excluded across all time points), if participants did not respond to at least 25% of stimuli (7 excluded at T1, 4 at T2, 4 at T3), or if hit rate (percent correctly identified repeat items) was below 10% (1 excluded at T1, 2 at T2). To increase measurement reliability, a parceling approach was used to analyze the MDT-OS data (Little et al., 2002, 2013; see Supplementary Methods for more details). Items were randomly assigned to three parcels, such that easy, medium, and difficult items (as assessed in Güsten et al., 2021) were evenly distributed across parcels. Within each of these parcels, corrected hit rate was calculated as hit rate minus false alarm rate (percent incorrect lure items) according to Snodgrass and Corwin (1988). In this way, three observed variables were created at each time point.

### 2.4 Statistical analysis

Statistical analyses were conducted using R (Version 4.3.0 [2023-04-21]; R Core Team, 2023) in RStudio (Version 2023.06.2 + 561; Posit Team, 2023) using *tidyverse* (Wickham et al., 2019).

First, univariate outlier detection was conducted on each observed variable at each time point, with data points further than 4 SDs from the mean being excluded (one data point: left ERC volume at T2). Next, multivariate outliers across time points were identified and excluded case-wise using the *faoutlier* R package with the classical product-moment method (criterion = 0.001; Chalmers & Flora, 2015). Six cases of subfield volume data were excluded (1 left CA1/CA2; 2 right CA3/DG; 1 right subiculum; 2 left ERC), as well as one case of VO_2_peak data (see Supplementary Figure S1).

Structural equation modeling (SEM) using the *OpenMx* package (Boker et al., 2021; Hunter, 2018; Neale et al., 2016; Pritikin et al., 2015) was used to investigate differences in change in hippocampal subfield volume and MDT-OS performance across training types, as well as to investigate associations between subfield volumes and MDT-OS performance, testing for bivariate associations. Observed variables were rescaled longitudinally, such that they had a mean of 0 and standard deviation at 1 at T1 to preserve the relationship between the same variables measured at different time points. The root mean square error of approximation (RMSEA) and the comparative fit index (CFI) were used to evaluate model fit, with RMSEA < 0.08 and CFI > 0.90 indicating acceptable model fit (Schermelleh-Engel et al., 2003). Model parameters were estimated using full information maximum likelihood (FIML; to account for missing data without the need for case-wise deletion) with bootstrapping (5,000 replications). Wald tests (*Z*-score calculated as a parameter estimate divided by its standard error) were used to test for statistical significance of parameters of interest (critical *Z* = 1.96). Likelihood-ratio tests (LRT) were used to check for factorial invariance across groups and time points and to test the assumption that change was equal across the first and second halves of the training when establishing the models.

**Fig. 5.**
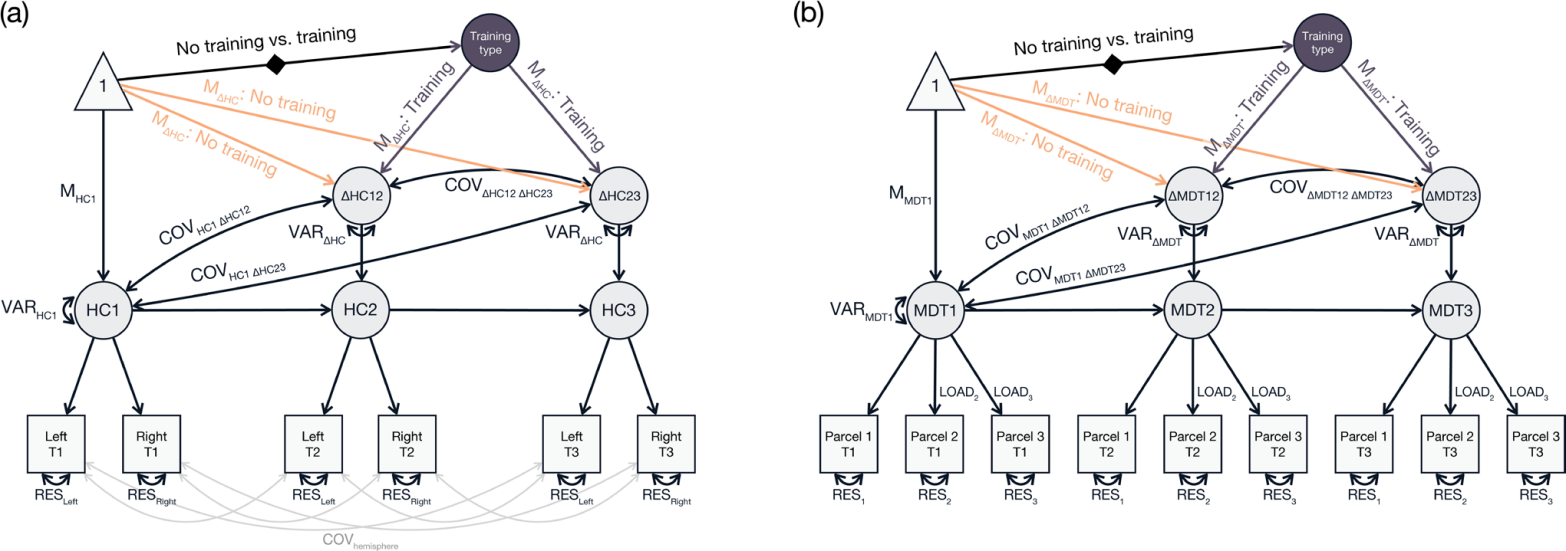
Latent change score models of (a) bilateral hippocampal subfield volume and (b) Mnemonic Discrimination Task for Objects and Scenes performance using a parcellation approach with training (language learning or exercise) as a moderating factor of change. HC = hippocampus, MDT = Mnemonic Discrimination Task, M = mean, COV = covariance, VAR = variance, RES = residual, LOAD = loading. Labeled paths are freely estimated, identically named paths are constrained to equal, and unlabeled paths are fixed to 1. Subfield volume models (a) were run separately for each subfield. The black diamond indicates a definition variable. Yellow indicates parameters estimated with data from participants not engaging in the respective training (coded as 0), and purple indicates parameters estimated with data from training participants (coded as 1).

Multivariate latent change score models (LCSM; McArdle & Hamagami, 2001; McArdle & Nesselroade, 1994; also see Kievit et al., 2018) were used to evaluate group differences in change in bilateral hippocampal subfield volume, with the right and left hemispheres of each subfield loading equally onto latent factors, and MDT-OS performance, with the three parcels loading onto latent factors at each time point. Each model was subject to tests of group invariance and invariance across time points to establish that longitudinal group comparisons were appropriate (Cheung & Rensvold, 1999; Widaman et al., 2010). See Figure 5 for visual representations and Supplementary Methods for more details regarding model set-up.

Baseline differences and the effects of language or exercise training on hippocampal subfield volume and MDT-OS performance were estimated using separate moderation models with binary definition variables, one set of models for language learning and one for aerobic exercise, and a stepwise approach to test for synergistic effects of the combined training. To this end, the mean of the variable of interest was estimated for those observations coded as 0 (no training), as well as the mean difference between observations coded as 0 and those coded as 1 (language or exercise). If a significant effect of language or exercise training was found, a follow-up analysis was conducted comparing the group who did the respective training only (LG or EG) to the L+EG to investigate whether combined language and exercise training has an additive effect above and beyond the effect of one training type alone. This was done by entering the data of training participants (two groups) into the same model with the binary definition variable coded as 0 for those who did not do the combined training and as 1 for those who did.

**Fig. 6.**
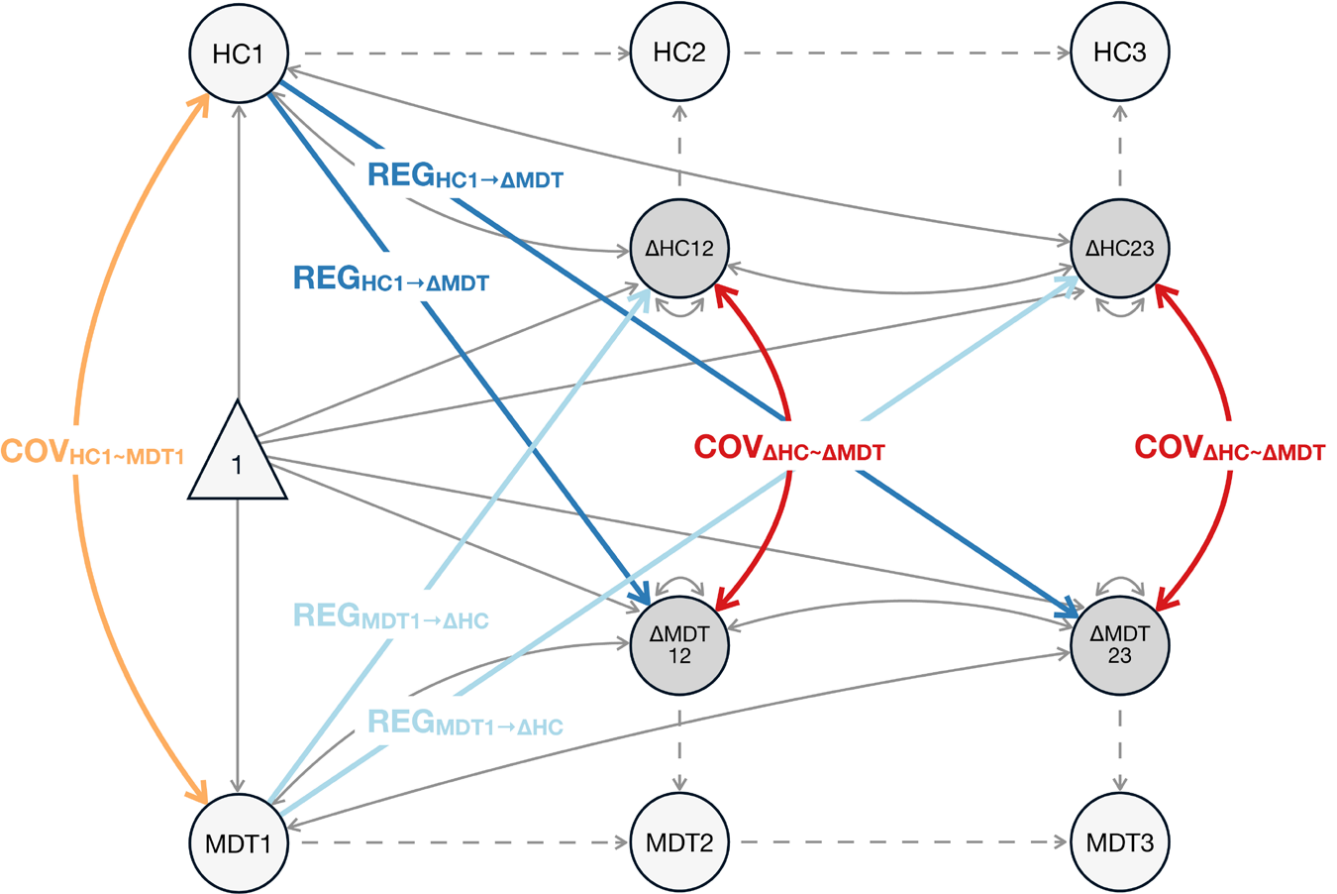
Bivariate latent change score model combining latent change score models of Mnemonic Discrimination Task for Objects and Scenes and bilateral hippocampal subfield volume to investigate bivariate associations. For the sake of visual clarity, this figure only shows the structural model (see Fig. 5 for the measurement models).

Finally, to investigate baseline–baseline, baseline–change, and change–change associations between hippocampal subfield volume and MDT-OS performance we used a bivariate LCSM (see Figure 6 for a visual representation of the structural model). Three correlational paths were included in this model: between hippocampal subfield volume and MDT-OS performance at T1, between latent changes from T1 to T2 and from T2 to T3, with change–change correlation estimates across timeframes constrained to be equal. Four regression paths were also included: one from hippocampal subfield volume at T1 to MDT-OS performance change from T1 to T2 and one to change from T2 and T3 (constrained to be equal), and one from MDT-OS performance change at T1 to hippocampal subfield volume change from T1 to T2 and one to change from T2 and T3 (constrained to be equal). Parameters were estimated for all groups combined. Testing of training effects and correlations between MDT-OS performance and hippocampal subfield volume was done separately due to the moderately sized sample (Kline, 2016).

Additionally, we ran a set of control analyses including covariates of no interest (age, sex, years of education) in the bivariate LCSM to test whether the relationships of interest would be attenuated by including demographic factors. Covariates were centered (M = 0, SD = 1) and included in each model, estimating the variance of each parameter, and the regression of each covariate onto baseline levels of hippocampal subfield volume and MDT-OS performance, as well as onto change in hippocampal subfield volume and MDT-OS performance (see Supplementary Fig. S3).

## 3 Results

In the current analyses, we used LCSMs with a moderation approach to test for the effects of foreign language learning and aerobic exercise on bilateral CA1/CA2, CA3/DG, subiculum, and ERC volume, as well as MDT-OS performance. The association between hippocampal subfield volume, particularly of the CA3/DG, and MDT-OS performance was tested using bivariate LCSMs.

Regarding sample characteristics, no differences were found across intervention groups in the number of fully compliant participants, age, sex, years of education, or DSST performance (see Table 1). Participants who exercised increased in physical fitness; those in the EG improved by 10.8%, SD = 14.3%, of their initial VO_2_peak, *t*_37_ = 4.63, Cohen’s *d* = .76, *p* < .001, and those in the L+EG improved by 10.1%, SD = 15.2%, *t*_24_ = 3.42, Cohen’s *d* = .66, *p* = .002, while participants who did not exercise showed no change in fitness; ACG: 3.7%, SD = 13.1%, *t*_29_ = 1.56, Cohen’s *d* = .28, *p* = .130, LG: 0.5%, SD = 11.6%, *t*_26_ = 0.23, Cohen’s *d* = .04, *p* = .823. There was no difference in change in fitness between the EG and the combined L+EG, *F*_1, 61_ = 0.03, *η*^2^ = 0.001, *p* = .858. Participants in the LG and the L+EG completed equivalent amounts of Spanish items (e.g., words, phrases, grammar exercises), including repetitions of the same items, in the Babbel application; LG: 9039 words, SD = 4212, L+EG: 7567, SD = 4734, *F*_1, 54_ = 1.51, η^2^ = 0.03.

**Table 1.** Descriptives of sample demographics, including a measure of cognition at T1, change in physical fitness, and words/phrases learned using the Babbel application.

	Group				Group difference statistic (χ*^2^* or *F*) and effect size (Cramér’s *V* or η^2^)
	Active Control	Language	Exercise	Language + Exercise	
*n* completed study, *N* = 142	35	34	40	33	
*n* fully compliant, *N* = 126	32	29	38	27	χ*^2^*_3, 142_ = 3.81, *V* = 0.16
Age at T1	70.5 ± 3.9	71.1 ± 3.6	69.8 ± 3.4	70.7 ± 3.6	*F*_3, 136_ = 0.84, η^2^ = 0.02
Sex, % females	40.0	58.8	50.0	51.5	χ*^2^*_3, 142_ = 2.49, *V* = 0.13
Years of education	13.4 ± 3.3	13.0 ± 3.3	13.2 ± 3.0	13.7 ± 3.4	*F*_3, 135_ = 0.35, η^2^ = 0.01
DSST score at T1	47.3 ± 8.6	48.9 ± 14.4	45.4 ± 10.8	50.3 ± 11.4	*F*_3, 121_ = 1.10, *η*^2^ = 0.03

*Note.* Mean and standard deviations reported unless otherwise indicated. DSST = Digit Symbol Substitution Task.

See Supplementary Table S4 for descriptive statistics of all observed variables, Supplementary Table S5 for their covariances at T1, and Supplementary Figure S2 for a visualization of all observed variables.

### 3.1 Hippocampal subfields

The LCSMs for bilateral CA1/CA2, subiculum, and ERC volume showed strict factorial invariance across both groups and time points. Strict factorial invariance over time could not be established for the CA3/DG model, that is, measurement variance was not stable across time. Descriptively, the right DG showed the highest residual variance (i.e., measurement error) at T3. Residual variance estimates were, therefore, allowed to vary across time points in models including CA3/DG. Fit indices were acceptable for all the models (see Supplementary Table S6).

#### 3.1.1 Testing for group differences at baseline

Language-learners (LG and L+EG) showed no difference from non-language learners (ACG and EG) in volume of any subfield at baseline. Exercisers (EG and L+EG) showed no baseline differences from non-exercisers (ACG and LG) in CA1/CA2 or in CA3/DG volume, but greater volume in the bilateral subiculum, *b* = 0.234, 95% CI [0.024, 0.444], and ERC, *b* = 0.227, 95% CI [0.027, 0.427] (see Supplementary Results regarding the interpretation of estimates; see Supplementary Tables S7–S10 for all parameter estimates). That is, at baseline, for every difference of an SD from the mean in the non-exercisers, a positive difference of 23.4% of an SD in subiculum volume (average SD across right and left = 55.0 mm^3^) and 22.7% in ERC volume (1 SD = 44.9 mm^3^) was expected in the exercisers.

#### 3.1.2 No main effect of time on subfield volume

Regarding linear change in hippocampal subfield volumes across all groups collapsed, no main effect of time was observed in any of the subfields (see Supplementary Tables S7–S10).

#### 3.1.3 Language learners show less CA3/DG volume decrease than non-language learners

**Fig. 7.**
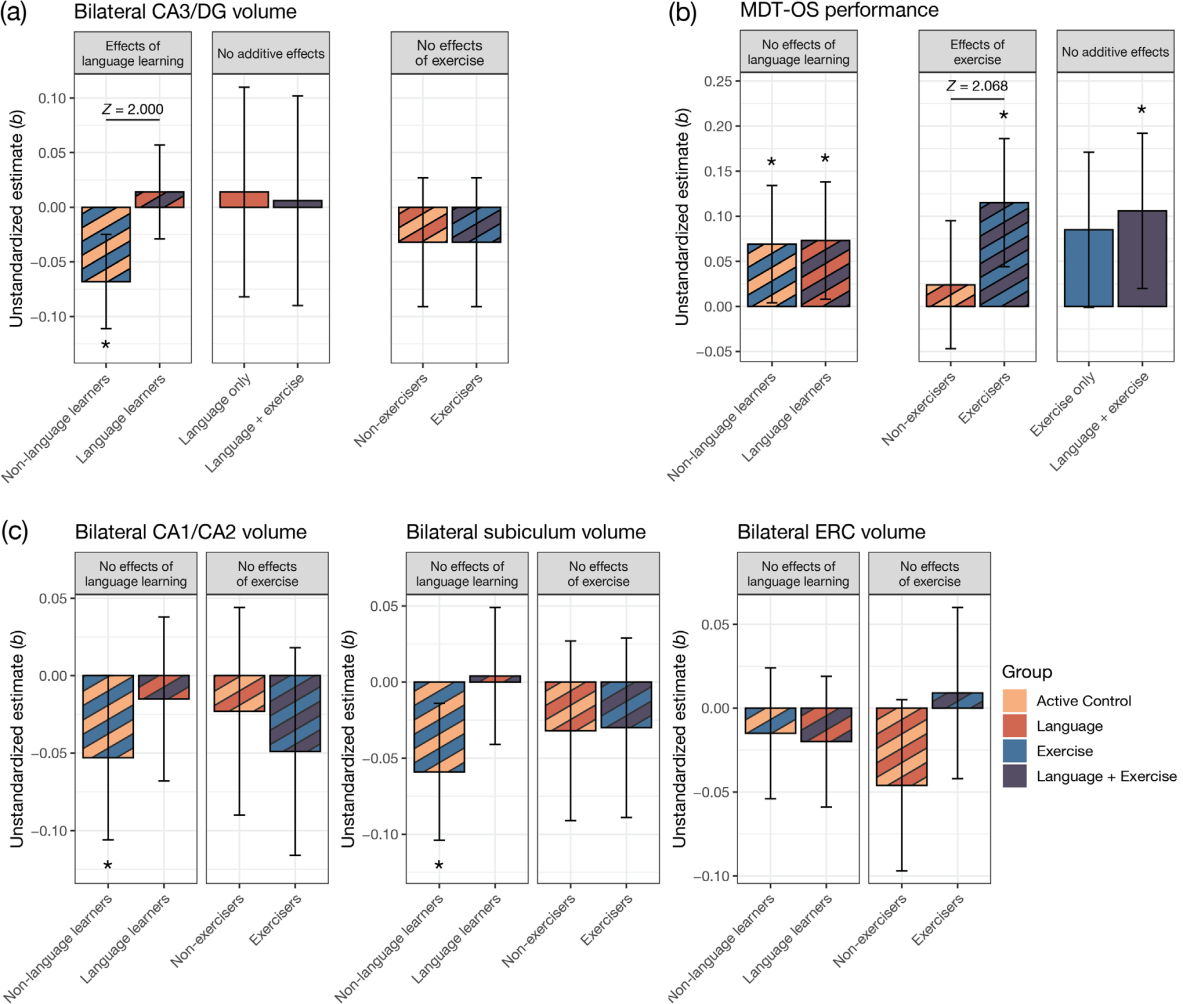
Positive effects of (a) language learning on bilateral CA3/DG volume and of (b) exercise on MDT-OS performance; no additive effects of combined training were observed. Neither language learning nor exercise had a significant positive effect on (c) volume in the CA1/CA2, subiculum, or ERC. Error bars indicate 95% confidence intervals. Solid lines across groups indicate a significant group difference at *Z* > 1.96. Non-language learners, non-exercisers, language-only, and exercise-only groups serve as a reference, therefore the comparison group estimates were calculated as the estimate of the reference group plus the estimate of the comparison group. *Significant difference from 0 indicated by the 95% confidence intervals.

Regarding training effects, there was a significant effect of language learning on bilateral CA3/DG volume, with non-language learners (ACG and EG) showing a significant decrease in volume, *b* = −0.068, 95% CI [−0.109, −0.027], and language learners (LG and L+EG) showing a significantly more positive change than non-language learners, *b* = 0.082, 95% CI [0.006, 0.158]. That is, every 3 months, the non-language learners decreased by 6.8% of an SD (1 SD = 45.9 mm^3^), while the difference in decrease between the non-language and language learners was 8.2% of an SD, with more negative change in non-language learners. There were no effects of language learning on any other subfields (CA1/CA2, subiculum, or ERC volume). Given the effect of language learning on CA3/DG volume, we investigated whether combined language and exercise training would be beneficial compared to language learning alone. No significant difference in change in CA3/DG volume in the L+EG compared to the LG was observed, *b =* −0.008, 95% CI [−0.149, 0.133] (see Fig. 7a).

No significant effect of aerobic exercise was found on volume of any subfield (see Supplementary Tables S7–S10).

### 3.2 MDT-OS performance

The LCSM for MDT-OS performance showed strict factorial invariance across both groups and time points, and fit indices were acceptable (see Supplementary Table S6).

#### 3.2.1 Testing for group differences at baseline

No baseline differences in MDT-OS performance were observed between language (LG and L+EG) and non-language groups (ACG and EG), or between exercise (EG and L+EG) and non-exercise groups (ACG and LG; see Supplementary Table S11 for all parameter estimates).

#### 3.2.2 Positive main effect of time on MDT-OS performance

A main effect of time on change in MDT-OS performance was observed, *b* = 0.068, 95% CI [0.015, 0.121], indicating an increase of 6.8% of an SD (1 SD = 0.197 CHR) in performance every 3 months across the whole sample.

#### 3.2.3 Exercisers show greater increase in MDT-OS performance than non-exercisers

We found no effect of language learning on change in MDT-OS performance (see Supplementary Table S11); non-language learners (ACG and EG) showed a significant increase in performance compared with zero, and language learners (LG and L+EG) showed no difference in change compared to non-language learners.

We found a significant effect of aerobic exercise on change in MDT-OS performance, with non-exercisers (ACG and LG) showing no significant change in MDT-OS performance, *b* = 0.022, 95% CI [−0.047, 0.091], and exercisers (EG and L+EG) showing significantly greater improvement than non-exercisers, *b* = 0.089, 95% CI [0.005, 0.173]. That is, every 3 months, the non-exercisers increased non-significantly by 2.2% of an SD, while the difference in change between non-exercisers and exercisers was 8.9% of an SD, with exercisers showing significantly more positive change. Given the effect of aerobic exercise on change in MDT-OS performance, we investigated whether combined language and exercise training would be beneficial compared to exercise alone. No significant difference in change in MDT-OS performance in L+EG compared to the EG was observed, *b* = 0.024, 95% CI [−0.094, 0.142] (see Fig. 7b).

### 3.3 Baseline subiculum volume predicts change in MDT-OS performance

All latent change variables showed statistically significant variance, therefore we next investigated associations between hippocampal subfield volume (each subfield in a separate model) and MDT-OS performance. All four models estimating baseline–baseline, baseline–change, and change–change associations showed acceptable fit indices (see Supplementary Table S6).

**Fig. 8.**
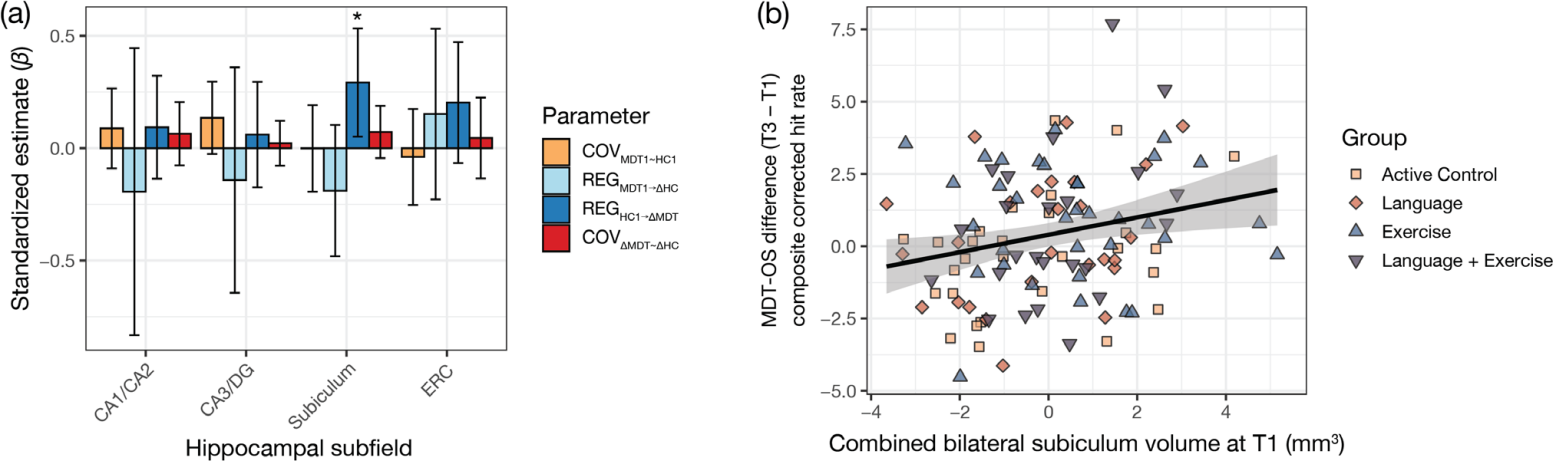
Subiculum volume at baseline predicted change in Mnemonic Discrimination Task for Objects and Scenes performance across the sample. (a) shows all standardized estimates for baseline–baseline, baseline–change, and change–change paths across models, and (b) visualizes the correlation between bilateral subiculum volume at T1 and change in MDT-OS from T1 to T3. CA = cornu ammonis, DG = dentate gyrus, ERC = entorhinal cortex, HC = hippocampus, MDT(-OS) = Mnemonic Discrimination Task (for Objects and Scenes), COV = covariance, REG = regression. Error bars represent 95% confidence intervals. Models for each subfield were estimated separately. Models were run on standardized data. Combined bilateral volumes and composite corrected hit rates were calculated for visualization in (b) only and were not used in analyses. *p < .050, indicating a parameter estimate significantly different from zero.

Regarding baseline–baseline associations, no significant correlations were found between volume in any of the subfields and MDT-OS performance at T1 (see Fig. 8a; see Supplementary Table S12 for all parameter estimates).

Regarding baseline–change associations, bilateral subiculum volume at T1 was positively associated with change in MDT-OS performance, *β* = 0.291, 95% CI [0.044, 0.538] (see Fig. 8). That is, for each SD difference in subiculum volume at baseline compared to the mean, a difference in change in MDT-OS performance of 29.3% of an SD was expected. Because we found a significant difference in change in MDT-OS performance between exercisers and non-exercisers, and also considering the baseline difference in subiculum volume between the two groups, we tested whether the association between baseline subiculum volume and MDT-OS change also differed across exercisers vs. non-exercisers *post hoc*. No statistically significant difference in this association between exercisers, *β* = 0.230, 95% CI [−0.058, 0.518], and non-exercisers, *β* = 0.353, 95% CI [0.065, 0.641], χ^2^_*df* = 1_ = 0.60, *p* = .440, was found.

Finally, no significant change–change associations were found between any of the subfield volumes and MDT-OS performance (see Fig. 8a).

#### 3.3.1 Demographic factors and cardiovascular fitness do not explain bivariate relationships

Finally, a set of control analyses including age, sex, and years of education as covariates in the bivariate LCSM was run to test whether the relationship between baseline subiculum volume and change in MDT-OS performance would be attenuated by including demographic factors (see Supplementary Fig. S3a). Fit indices of all models were acceptable, RMSEAs ≤ .039, CFIs ≥ .956. Age negatively predicted volume of all subfields at T1; CA1/CA2: *β* = −0.283, 95% CI [−0.452, −0.114], CA3/DG: *β* = −0.170, 95% CI [−0.335, −0.005], subiculum: *β* = −0.226, 95% CI [−0.381, −0.071], and ERC: *β* = −0.234, 95% CI [−0.403, −0.065]. That is, for each SD older a participant was compared to the mean (ca. 3.5 years), subfield volumes besides the CA3/DG were expected to be 17.0% to 28.3% of an SD smaller. Age did not predict changes in volume in any subfield. Neither sex nor years of education predicted volume at T1 or change in volume in any subfield. Age also predicted baseline MDT-OS performance, *β*s = −0.238 to −0.230, 95% CIs [−0.387 to −0.375, −0.092 to −0.085]. That is, for each SD older a participant was, a score of 23.8% to 23.0% of an SD worse was expected. Age was not related to change in MDT-OS performance over time. Neither sex nor years of education predicted baseline levels or change in MDT-OS performance. Including age, sex, and years of education in the model did not affect the relationship found between subiculum volume at T1 and change in MDT-OS performance, *β* = 0.294, 95% CI [0.057, 0.531].

Finally, since a main effect of exercise was found on change in both fitness and MDT-OS performance, and although we found that the relationship found between baseline subiculum volume and change in MDT-OS performance was observed to be independent of exercise training, we tested whether including VO_2_peak in the model would mediate the relationship between baseline subiculum volume and change in MDT-OS performance. Physical fitness (indexed with VO_2_peak) was therefore included in the model *post hoc* to examine whether this association would remain significant. To this end, a pseudo-LCSM of fitness was used to estimate change in VO_2_peak. Baseline–baseline and change–change correlation paths as well as baseline–change regression paths were estimated (see Supplementary Fig. S3b). Fit indices of all models were acceptable, RMSEA ≤ .029, CFI ≥ .982. Neither fitness at T1 nor change in fitness was significantly associated with baseline levels or changes in hippocampal subfield volume or MDT-OS performance. Including fitness in the model did not account for the relationship between subiculum volume at T1 and change in MDT-OS performance, *β* = 0.299, 95% CI [0.066, 0.532].

A set of sensitivity analyses was run to rule out the influence of outlier removal and compliance. These are described in the Supplementary Results.

## 4 Discussion

Our study explored the effects of 6 months of foreign language acquisition and aerobic exercise on hippocampal subfield volume and performance in a mnemonic discrimination task among healthy, previously sedentary older adults aged 63 to 78 years. We implemented online and at-home language learning and aerobic exercise programs, which could be easily incorporated into participants’ daily routines and required minimal supervision. This approach contrasts with in-lab protocols that necessitate intensive immersive learning or multiple in-person intervention sessions per week (e.g., Cui et al., 2023; Erickson et al., 2011; Mårtensson et al., 2012). Our findings indicate that foreign language learning can support the attenuation of CA3/DG atrophy, and that aerobic exercise can enhance performance in a mnemonic discrimination task. Interestingly, while the combined language and exercise training yielded benefits in terms of both CA3/DG volume and mnemonic discrimination, we did not observe any boosted outcomes from the combined intervention. Additionally, we discovered that a larger subiculum volume at baseline was associated with a more significant improvement in mnemonic discrimination across all training groups over the course of the study. However, we found no associations between changes in subfield volume and changes in mnemonic discrimination.

### 4.1 Foreign language learning is associated with maintenance of CA3/DG volume

Our finding that language learners, compared to non-language learners, maintained their CA3/DG volume aligns with prior research exploring the impact of foreign language learning on hippocampus volume in young adults. For instance, a 1.4% increase in gray matter volume within a cluster of voxels in the right hippocampus was reported following 10 weeks of foreign vocabulary learning (Bellander et al., 2016). Similarly, intensive interpreter training (zero to fluency in 10 months) led to bilateral hippocampal volume increases (hippocampal volumes pre- and post-training were not reported so the relative magnitude of change is unknown; Mårtensson et al., 2012). However, in older adults, an 11-week intervention study found no evidence of language learning affecting hippocampal volume (Nilsson et al., 2021). Contrary to this, our 6-month intervention appears to have protected the CA3/DG from the volume loss observed in non-language learners.

The structural integrity of the CA3/DG has been linked to associative memory in aging humans, with smaller CA3/DG volumes correlating with worse associative memory performance (Bender et al., 2013; Shing et al., 2011). The language learning approach used in the Babbel application, which was employed in the current study, relies heavily on associative memory of words or phrases in German and Spanish (e.g., Hallo! = ¡Hola!). This repeated engagement of associative memory during the learning of new Spanish vocabulary and phrases may have contributed to the preservation of CA3/DG volume in our Spanish-learning participants. This hypothesis is further supported by the findings of Nilsson et al. (2021), who observed that initial associative memory ability positively predicted vocabulary proficiency at the end of their 11-week intervention, suggesting that older adults utilize associative memory in the acquisition of vocabulary in a new language. Collectively, our finding suggests that prolonged periods of foreign language acquisition may help protect older adults against volume decline specifically in the CA3/DG, potentially through the increased engagement of associative memory processes during vocabulary acquisition.

### 4.2 No benefits of aerobic exercise on hippocampal subfield volume

Contrary to our initial hypothesis, we found no evidence suggesting an impact of aerobic exercise on the volume of hippocampal subfields, particularly the CA3/DG. Past research examining the influence of exercise on hippocampal volume has yielded diverse outcomes. Erickson et al. (2011) were the first to observe exercise intervention-induced changes in hippocampal volume, showing a 2% increase in anterior hippocampal volume after 1 year. Regarding individual subfields, Frodl et al. (2020) observed a positive change in the bilateral CA4/DG following a 16-week period among young and middle-aged adults; volume increased by 1.1% in exercising participants, while non-exercising participants experienced a decrease of 2.5% (Frodl et al., 2020). In contrast, a study carried out on older adults using high-resolution imaging on a 7T scanner found no impact of physical activity on DG volume after 24 months of exercise (Rosano et al., 2017), though an exercise effect on left hippocampus volume was observed. One potential explanation could be that the benefits of aerobic exercise on CA3/DG are only apparent if the exercise regime is implemented starting in middle age. This hypothesis may be explored in future studies, specifically focusing on the optimal time for sedentary adults to initiate exercise to preserve CA3/DG volume during aging. Another key difference between studies was the hippocampal segmentation approaches. Indeed, even within the same sample, Frodl et al. (2020) found that using an automated segmentation approach on T1-weighted images versus using a manual tracing approach on high-resolution hippocampal images resulted in divergent results; namely, they only saw exercise effects on CA4/DG volume using the former. Altogether, this could suggest that aerobic exercise has a more holistic effect on the hippocampus and surrounding structures, rather than a targeted effect on the DG.

This study also differs from previous studies investigating the effect of exercise on hippocampal structure in that participants were not supervised during their aerobic exercise sessions. While this approach offers some ecological validity regarding the accessibility of unsupervised exercise, it also increases variability in the intensity of exercise performed by participants. Indeed, exercise intensity may be an important factor for hippocampal structure-related outcomes. For example, two separate 26-week studies including older adults with mild cognitive impairment investigated the effect of aerobic exercise on hippocampal volume: one study increased the exercise intensity to 70–80% of one’s age-specific target heart rate (or heart rate reserve; HRR) within the first 12 weeks of the study (ten Brinke et al., 2015), while the other gradually increased the target heart rate to 60–75% HRR over the course of the study (Morris et al., 2017). Indeed, the former study found significantly more positive changes in hippocampal structure in those who exercised, while the latter did not (Morris et al., 2017; ten Brinke et al., 2015). Future studies could consider monitoring the individualized intensity of exercise sessions (e.g., using heart rate, which was measured in the current study but not used to adjust difficulty; Frodl et al., 2020) and encouraging participants to reach an optimal intensity zone (over 70% HRR). This approach may better effectively promote the preservation of hippocampal subfield structural integrity. Finally, the current exercise intervention lasted approximately 6 months, which is notably shorter than the 12- and 24-month studies conducted by Erickson et al. (2011) and Rosano et al. (2017). While interventions of longer than 24 weeks (ca. 5.5 months) were found to have a significant positive effect on total hippocampal volume (Wilckens et al., 2021), it may take longer to observe exercise-related effects in the subfield volumes of healthy older adults. However, Rosano et al. (2017) also observed no subfield-specific exercise effects when controlling for baseline volumes, even after 24 months.

### 4.3 No benefits of foreign language learning on mnemonic discrimination

Despite the observed maintenance effect of language learning on CA3/DG volume and the established reliance of pattern separation on the DG (Berron et al., 2016), we did not find any differential behavioral effects. Both the non-language learners and language learners demonstrated similar improvements in mnemonic discrimination during the intervention. This finding aligns with a previous intervention conducted on young adults, where no improvement in mnemonic discrimination was observed after foreign vocabulary learning (Bellander et al., 2016). Our current implementation of foreign language learning may not specifically target mnemonic discrimination as assessed with the MDT-OS and instead might be more related to associative memory or episodic memory in a broader sense. Indeed, in a set of analyses performed on the same sample, we found effects of language learning on a latent factor of episodic memory. This factor comprised two independent tasks of associative and visuospatial memory (Wenger et al., *in preparation*). It is plausible that engaging in foreign language vocabulary acquisition and interacting with new syntactical structures triggers more general episodic memory processes. This may explain the observed maintenance of hippocampal subfield volumes, but it does not seem to contribute to specific improvements in mnemonic discrimination ability.

### 4.4 Moderate aerobic exercise improves mnemonic discrimination

The present analyses revealed improved mnemonic discrimination among exercisers in comparison to sedentary older adults. This finding complements the growing body of cross-sectional evidence suggesting that a single bout of exercise can enhance mnemonic discrimination in older adults compared to a no-exercise condition (Callow et al., 2023), and that regular self-reported exercise predicts improved performance in mnemonic discrimination tasks in middle-aged adults (Bernstein & McNally, 2019). It also corroborates the results of a 16-week exercise (combined with video game) training study, where aerobic exercise was found to be beneficial for mnemonic discrimination (Cui et al., 2023). This is also consistent with a recent systematic review and meta-analysis of randomized controlled trials involving healthy older adults, which found that participation in aerobic exercise leads to improvements in episodic memory (Aghjayan et al., 2022; but also see Barha et al., 2017; Kelly et al., 2014; Young et al., 2015). In their meta-analyses, Aghjayan et al. (2022) highlighted that the most effective interventions were those that lasted between 18 and 39 weeks and involved three weekly exercise sessions lasting 15 to 90 minutes each (also see Düzel et al., 2016). This aligns with our current 6 month-long intervention (approximately 26 weeks), consisting of 30- to 50-minute sessions conducted three to four times a week, which resulted in exercise-related improvements in mnemonic discrimination.

The mechanisms responsible for the impact of exercise on episodic memory and mnemonic discrimination remain elusive. Several potential factors are under investigation (Augusto-Oliveira et al., 2023), including neurotrophic factors such as brain-derived neurotrophic factor (BDNF) and neuromodulatory systems such as the cholinergic system. Peripheral BDNF levels are known to increase in response to exercise bouts in older adults (Dinoff et al., 2017; Håkansson et al., 2016), though findings are less consistent regarding the effect of long-term exercise (Tarassova et al., 2020; also see Szuhany et al., 2015 for a meta-analytic review). Moreover, BDNF in the DG has been implicated in successfully encoding similar but not identical spatial representations in rats (Bekinschtein et al., 2013). A cross-sectional study found that the relationship between physical fitness and mnemonic discrimination in young adults was moderated by serum BDNF levels (Whiteman et al., 2014). However, the longitudinal associations of BDNF levels, exercise, fitness, and mnemonic discrimination in older adults have not yet been explored (see Voss et al., 2019 for a discussion on the role of BDNF in the association between physical activity and memory; see Supplementary Results for null results regarding BDNF in the current sample). In terms of neurotransmitters, both individual exercise bouts and long-term exercise have been found to benefit cholinergic input to the rat hippocampus (see Zong et al., 2022). Acetylcholine levels in the hippocampus appear to play a crucial role in pattern separation versus pattern completion, with higher levels associated with pattern separation and encoding and lower levels with pattern completion and retrieval (Decker & Duncan, 2020). Regular exercise could potentially increase cholinergic input to the hippocampus, thereby enhancing pattern separation and thus mnemonic discrimination.

### 4.5 Combined language and exercise leads to benefits in terms of both CA3/DG volume and MDT-OS performance but not to boosted outcomes

**Fig. 9.**
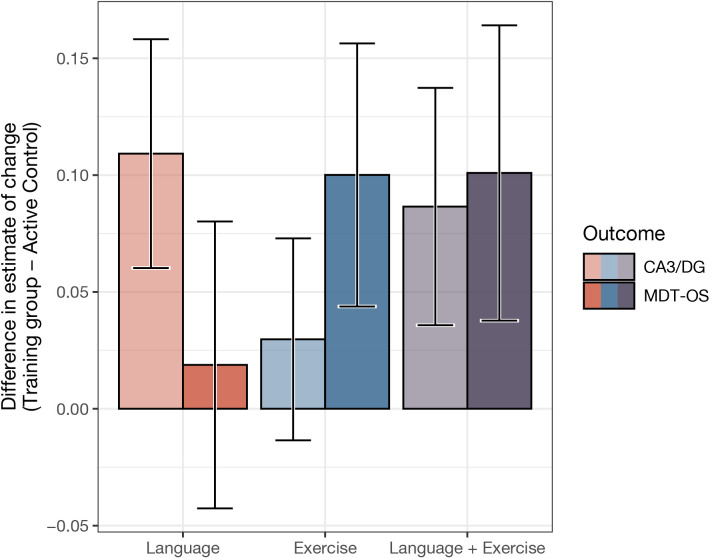
Estimates of change in CA3/DG volume and MDT-OS in each of the training groups compared to the active control group. The language-only group benefited only in terms of CA3/DG volume and the exercise-only group benefited only in terms of MDT-OS performance, while the combined training group showed positive changes compared to the active control group on both outcome variables. Error bars depict standard errors.

Even though all participants who engaged in foreign language learning demonstrated maintenance of CA3/DG volume compared to those who did not, though participants who both learned a foreign language and exercised did not show a greater benefit in terms of CA3/DG volume. Likewise, while all participants who performed aerobic exercise showed greater improvements on the MDT-OS task than those who did not, no significant difference was observed between participants who only exercised and those who both learned Spanish and exercised regularly. This indicated that individuals who engaged in both types of training showed benefits associated with language learning and aerobic exercise, demonstrated by positive changes in both CA3/DG volume (maintenance) and MDT-OS performance (increase) compared to the active control group (see Fig. 9). That is, those who did both language learning and aerobic exercise showed complementary benefits from each training type, suggesting that combination training is most beneficial when considering both observed outcomes. However, there was no evidence to support the hypothesis that combined cognitive and physical training would lead to boosted outcomes, particularly regarding cognitive function; the effect of the intervention on MDT-OS performance was comparable between the exercise-only and combined groups.

Past studies investigating combined cognitive and physical training have shown mixed results regarding boosted cognitive outcomes for combined training groups compared to single training-type groups. Three studies have shown positive results: Oswald et al. (2006) found that older adults who participated in both cognitive and physical training for 5 years showed greater improvements in a composite of cognitive function than those in a cognitive-only training group. Likewise, Fabre et al. (2002) reported that a combined training group significantly improved in a composite score of memory tasks more than either of the single groups after 2 months of training. Finally, Cui et al. (2023) found that participants who navigated in a 3D open-world video game while cycling significantly improved in mnemonic discrimination, which was significantly greater than the improvement in the video game-only group or the exercise-only group. However, Legault et al. (2011) found no effects of 4 months of any training type on an episodic memory composite, even when the training types were combined.

A single bout of aerobic exercise induces a cascade of events including neurogenesis and increased synaptic plasticity (Biedermann et al., 2016; van Praag, 2008). Engaging in a cognitively challenging activity during or shortly after exercise can enhance neuron survival and strengthen connectivity, thus improving behavioral outcomes. This led us to hypothesize that cognitive and physical training would act synergistically in the current combined group, inducing greater benefits than either training alone. This concept, termed “guided plasticity facilitation” by Fissler et al. (2013), involves physical exercise facilitating plasticity by increasing cell proliferation and long-term potentiation, and cognitive activity guiding plasticity by regulating synaptic changes. In the present study, in order to maintain motivation and adherence, participants were free to choose when they exercised and when they learned Spanish. However, it may be necessary to always exercise shortly before or while engaging in a cognitively demanding activity such as language learning, to see an additive effect on hippocampal structure or mnemonic discrimination. This has yet to be conclusively shown; Nilsson et al. (2020) found no evidence that exercising before performing working memory training was more beneficial for a cognitive composite than *vice versa*, while Cui et al. (2023) reported greater improvements in mnemonic discrimination when participants completed cognitive and physical training simultaneously. Interestingly, Birdsell (2023) showed that exercising before language learning can have a positive impact on long-term foreign vocabulary retention.

Finally, the combined group in the current study spent the same amount of time training as both the language- and exercise-only groups overall, but less time on the individual training regimes, language learning: *t*_54_ = −2.16, *p* = .035, exercise: *t*_63_ = −3.20, *p* = .002. It is, therefore, notable that the observed effects were comparable between the single training-type groups and the combined group. Therefore, the dosage within the combined group may have been insufficient to amplify beneficial outcomes, though it was sufficient to induce both main effects of the respective training types. Notably, the combined group still completed more than the recommend dose for episodic memory effects discussed by Aghjayan et al. (2022). Additionally, the current language-learning intervention had a similar intensity to previous interventions (albeit with null results; Berggren et al., 2020; Nilsson et al., 2021), with weekly classes supplemented by at-home learning sessions. Additionally, the assigned training sessions did not differ between the groups; the LG and L+EG were both assigned the same amount of language learning while the EG and the L+EG were both assigned the same amount of aerobic exercise. Future studies should, therefore, consider the feasibility of such training programs; intense training regimes involving 1.5 hours of multiple types of training 3–4 days a week may be challenging to implement in older populations.

Overall, the observed pattern of results suggests that language learning and aerobic exercise work simultaneously to produce co-occurring and complementary benefits for both brain and behavior. These training types may target partially distinct yet interdependent mechanisms, such that their combination is necessary to induce measurable changes at both the structural (CA3/DG volume) and the behavioral level (mnemonic discrimination). While these observed changes were not greater for the combined group, this does not entirely preclude synergistic effects of combined training. For example, there may be downstream effects that are only induced in the presence of both structural and behavioral effects, such as performance on a more general memory test. The combined intervention may not amplify each individual effect in isolation but rather facilitate a coupling between the structural plasticity and functional outcomes such that downstream effects are enabled. Additionally, in terms of outcomes, the two training types did not appear to interfere with one another; the combined group did not show reduced benefits on either CA3/DG volume or mnemonic discrimination performance. Ultimately, the current data do not provide definitive evidence for or against a synergistic interaction between language learning and aerobic exercise. Future studies with larger sample sizes and increased statistical power will be necessary to more conclusively determine whether the observed co-occurrence of structural and behavioral changes, indeed, reflects synergistic mechanisms between physical exercise and cognitive training.

### 4.6 Baseline subiculum volume predicts mnemonic discrimination improvement

Interestingly, we found that the volume of the subiculum at baseline predicted improvement on the MDT-OS, irrespective of the training conducted. This finding was somewhat unexpected, considering the well-established role of the CA3 and DG, but not the subiculum, in pattern separation. Cross-sectional studies using functional MRI have consistently identified the CA3/DG as being uniquely activated during tasks that recruit pattern separation as opposed to pattern completion (Bakker et al., 2008; Berron et al., 2016; Lacy et al., 2011; Yassa et al., 2010). However, our results indicate that neither the baseline volume nor the change in volume of the CA3/DG subfield significantly predicted change in mnemonic discrimination. It is plausible that regional activation of the CA3/DG, rather than its volume, is the key factor predicting performance in mnemonic discrimination. Perhaps participants who improved in the current task would also show greater activation in the CA3/DG while performing the task. Future longitudinal research might consider including task-based functional MRI in addition to structural MRI to examine changes in activation in the CA3/DG over time.

Importantly, in the current study, participants were presented with identical stimuli at each time point. The finding that baseline subiculum volume positively predicted change in MDT-OS performance over time suggests that the subiculum may play a role in mnemonic discrimination, specifically across repeated trials over a longer period. That is, baseline subiculum volume may support increased practice effects via greater memory recall at later time points for repeated items. This is supported by rodent work showing that optogenetically inhibiting the subiculum during memory recall impairs performance in episodic-like memory tasks (Ledergerber & Moser, 2017; Roy et al., 2017). In cross-sectional MRI studies in humans, increased activity in the subiculum has been observed during memory retrieval (Eldridge et al., 2005; Gabrieli et al., 1997; Zeineh et al., 2003), and greater volume of the subiculum has been positively associated with performance on delayed recall tasks (Zammit et al., 2017). Future research may consider using parallel versions of mnemonic discrimination tasks to disentangle delayed memory recall across longer periods (i.e., practice effects) and changes in mnemonic discrimination itself. Of note, practice effects, often regarded as a nuisance factor in longitudinal studies, may be a useful tool for predicting cognitive decline in aging. Indeed, reduced practice effects have been associated with cognitive status (e.g., mild cognitive impairment or dementia vs. healthy cognition) and future cognitive decline, as well as certain risk factors for Alzheimer’s disease, the most common cause of dementia (see Jutten et al., 2020 for a review). To date, little research has explored the effects of cognitive and physical exercise interventions practice effects specifically, rather than improvements in cognition *per se*.

### 4.7 Limitations

It is important to recognize several limitations in the current study that should be considered when interpreting the results. Firstly, as a longitudinal study involving active interventions, our sample size was moderate, albeit comparable to similar studies. We recruited only healthy older adults who were not fluent in more than two languages (German and one other language) and who had previously led a sedentary lifestyle. This group may possess other genetic or lifestyle factors that protect them from cognitive decline and impairment later in life. The generalizability of the current findings should be further investigated in future studies recruiting from more diverse populations as well as clinical populations. Regarding the statistical analyses reported here, a parceling approach was used to identify a latent factor of mnemonic discrimination at each time point. Ideally, multiple different tasks that all require mnemonic discrimination should be completed by participants to estimate a more valid latent factor of this cognitive function. Finally, strict factorial invariance could not be established for the CA3/DG model, and residual variances were therefore permitted to vary across time points. While this approach appropriately accounts for measurement instability, it means that latent CA3/DG volumes may not reflect the exact same measure at all time points, and our finding that language learning preserved CA3/DG volume should be interpreted with this limitation in mind.

### 4.8 Conclusion

In this set of analyses, we examined the effects of online foreign language learning and at-home aerobic exercise on hippocampal subfield volume and mnemonic discrimination, a crucial aspect of episodic memory, in older adults. Our findings suggest that both foreign language learning and aerobic exercise individually complementary confer benefits against age-related declines. Language training appears to protect against loss of CA3/DG volume, while aerobic exercise enhances mnemonic discrimination. We did not find evidence to suggest that combining language learning and aerobic exercise leads to greater benefits compared to either training alone. Rather, the combined intervention was the only condition in which both structural and behavioral benefits were observed concurrently. These effects may become functionally relevant when considered jointly; for example, the presence of both structural and behavioral changes may facilitate improvements in other cognitive outcomes that would otherwise not be observed. Future research will be needed to understand whether language learning and aerobic exercise act synergistically to induce effects on general and ecologically valid measures of cognitive functioning.

## Supplementary Material

Supplementary Material

## Data Availability

Data and code are available on OSF (view-only link: https://osf.io/mz83w/?view_only=2c197ee180604db3aaae59808dd21044).

## Footnotes

1 Approximately half of the sample was part of the longitudinal Berlin Aging Study II (BASE-II), which offers a rich set of additional variables such as socioeconomic background, as well as extensive medical and genetic data.

2
http://shared-reading.de/

## References

[IMAG.a.1306-b1] Aghjayan, S. L., Bournias, T., Kang, C., Zhou, X., Stillman, C. M., Donofry, S. D., Kamarck, T. W., Marsland, A. L., Voss, M. W., Fraundorf, S. H., & Erickson, K. I. (2022). Aerobic exercise improves episodic memory in late adulthood: A systematic review and meta-analysis. Communications Medicine, 2(1), 15. 10.1038/s43856-022-00079-7 35603310 PMC9053291

[IMAG.a.1306-b2] Antoniou, M., & Wright, S. M. (2017). Uncovering the mechanisms responsible for why language learning may promote healthy cognitive aging. Frontiers in Psychology, 8, 2217. 10.3389/fpsyg.2017.02217 29326636 PMC5736569

[IMAG.a.1306-b3] Augusto-Oliveira, M., Arrifano, G. P., Leal-Nazaré, C. G., Santos-Sacramento, L., Lopes-Araújo, A., Royes, L. F. F., & Crespo-Lopez, M. E. (2023). Exercise reshapes the brain: Molecular, cellular, and structural changes associated with cognitive improvements. Molecular Neurobiology, 60(12), 6950–6974. 10.1007/s12035-023-03492-8 37518829

[IMAG.a.1306-b4] Bakker, A., Kirwan, C. B., Miller, M., & Stark, C. E. L. (2008). Pattern separation in the human hippocampal CA3 and dentate gyrus. Science, 319(5870), 1640–1642. 10.1126/science.1152882 18356518 PMC2829853

[IMAG.a.1306-b5] Bamidis, P. D., Vivas, A. B., Styliadis, C., Frantzidis, C., Klados, M., Schlee, W., Siountas, A., & Papageorgiou, S. G. (2014). A review of physical and cognitive interventions in aging. Neuroscience & Biobehavioral Reviews, 44, 206–220. 10.1016/j.neubiorev.2014.03.019 24705268

[IMAG.a.1306-b6] Barha, C. K., Davis, J. C., Falck, R. S., Nagamatsu, L. S., & Liu-Ambrose, T. (2017). Sex differences in exercise efficacy to improve cognition: A systematic review and meta-analysis of randomized controlled trials in older humans. Frontiers in Neuroendocrinology, 46, 71–85. 10.1016/j.yfrne.2017.04.002 28442274

[IMAG.a.1306-b7] Bassett, D. R., & Howley, E. T. (2000). Limiting factors for maximum oxygen uptake and determinants of endurance performance. Medicine & Science in Sports & Exercise, 32(1), 70–84. 10.1097/00005768-200001000-00012 10647532

[IMAG.a.1306-b8] Baumeister, H., Gellersen, H. M., Polk, S. E., Lattmann, R., Wuestefeld, A., Wisse, L. E. M., Glenn, T., Yakupov, R., Stark, M., Kleineidam, L., Roeske, S., Morgado, B. M., Esselmann, H., Brosseron, F., Ramirez, A., Lüsebrink, F., Synofzik, M., Schott, B. H., Schmid, M. C.,…for the DELCODE study group. (2025). Disease stage–specific atrophy markers in Alzheimer’s disease. Alzheimer’s & Dementia, 21(7), e70482. 10.1002/alz.70482 PMC12279471 40691867

[IMAG.a.1306-b9] Bekinschtein, P., Kent, B. A., Oomen, C. A., Clemenson, G. D., Gage, F. H., Saksida, L. M., & Bussey, T. J. (2013). BDNF in the dentate gyrus is required for consolidation of “pattern-separated” memories. Cell Reports, 5(3), 759–768. 10.1016/j.celrep.2013.09.027 24209752 PMC3898274

[IMAG.a.1306-b10] Bellander, M., Berggren, R., Mårtensson, J., Brehmer, Y., Wenger, E., Li, T.-Q., Bodammer, N. C., Shing, Y.-L., Werkle-Bergner, M., & Lövdén, M. (2016). Behavioral correlates of changes in hippocampal gray matter structure during acquisition of foreign vocabulary. NeuroImage, 131, 205–213. 10.1016/j.neuroimage.2015.10.020 26477659

[IMAG.a.1306-b11] Bender, A. R., Daugherty, A. M., & Raz, N. (2013). Vascular risk moderates associations between hippocampal subfield volumes and memory. Journal of Cognitive Neuroscience, 25(11), 1851–1862. 10.1162/jocn_a_00435 23767922

[IMAG.a.1306-b12] Bender, A. R., Keresztes, A., Bodammer, N. C., Shing, Y. L., Werkle-Bergner, M., Daugherty, A. M., Yu, Q., Kühn, S., Lindenberger, U., & Raz, N. (2018). Optimization and validation of automated hippocampal subfield segmentation across the lifespan. Human Brain Mapping, 39(2), 916–931. 10.1002/hbm.23891 29171108 PMC5861710

[IMAG.a.1306-b13] Bennett, I. J., Stark, S. M., & Stark, C. E. L. (2019). Recognition memory dysfunction relates to hippocampal subfield volume: A study of cognitively normal and mildly impaired older adults. The Journals of Gerontology: Series B, 74(7), 1132–1141. 10.1093/geronb/gbx181 PMC6748802 29401233

[IMAG.a.1306-b14] Berggren, R., Nilsson, J., Brehmer, Y., Schmiedek, F., & Lövdén, M. (2020). Foreign language learning in older age does not improve memory or intelligence: Evidence from a randomized controlled study. Psychology and Aging, 35(2), 212–219. 10.1037/pag0000439 32011156

[IMAG.a.1306-b15] Bernstein, E. E., & McNally, R. J. (2019). Examining the effects of exercise on pattern separation and the moderating effects of mood symptoms. Behavior Therapy, 50(3), 582–593. 10.1016/j.beth.2018.09.007 31030875

[IMAG.a.1306-b16] Berron, D., Neumann, K., Maass, A., Schütze, H., Fliessbach, K., Kiven, V., Jessen, F., Sauvage, M., Kumaran, D., & Düzel, E. (2018). Age-related functional changes in domain-specific medial temporal lobe pathways. Neurobiology of Aging, 65, 86–97. 10.1016/j.neurobiolaging.2017.12.030 29454154

[IMAG.a.1306-b17] Berron, D., Schutze, H., Maass, A., Cardenas-Blanco, A., Kuijf, H. J., Kumaran, D., & Düzel, E. (2016). Strong evidence for pattern separation in human dentate gyrus. Journal of Neuroscience, 36(29), 7569–7579. 10.1523/JNEUROSCI.0518-16.2016 27445136 PMC6705559

[IMAG.a.1306-b18] Biedermann, S. V., Fuss, J., Steinle, J., Auer, M. K., Dormann, C., Falfán-Melgoza, C., Ende, G., Gass, P., & Weber-Fahr, W. (2016). The hippocampus and exercise: Histological correlates of MR-detected volume changes. Brain Structure and Function, 221(3), 1353–1363. 10.1007/s00429-014-0976-5 25550000

[IMAG.a.1306-b19] Birdsell, B. J. (2023). Exercising before learning enhances long-term memory for foreign language vocabulary and improves mood. Journal for the Psychology of Language Learning, 5(1), e5112123. 10.52598/jpll/5/1/3

[IMAG.a.1306-b20] Boker, S. M., Neale, M. C., Maes, H. H., Wilde, M. J., Spiegel, M., Brick, T. R., Estabrook, R., Bates, T. C., Mehta, P., von Oertzen, T., Gore, R. J., Hunter, M. D., Hackett, D. C., Karch, J., Brandmaier, A. M., Pritikin, J. N., Zahery, M., Kirkpatrick, R. M., Wang, Y., & Niesen, J. (2021). OpenMx 2.19.6 User Guide. 10.32614/cran.package.openmx

[IMAG.a.1306-b21] Bosquet, L., Léger, L., & Legros, P. (2002). Methods to determine aerobic endurance. Sports Medicine, 32(11), 675–700. 10.2165/00007256-200232110-00002 12196030

[IMAG.a.1306-b22] Braumann, K.-M., Ziegler, M., & Reer, R. (2004). FREIZEIT- UND FITNESSSPORT. Sports Orthopaedics and Traumatology Sport-Orthopädie – Sport-Traumatologie, 20(2), 71–75. 10.1078/0949-328X-00199

[IMAG.a.1306-b23] Burke, S. N., Wallace, J. L., Nematollahi, S., Uprety, A. R., & Barnes, C. A. (2010). Pattern separation deficits may contribute to age-associated recognition impairments. Behavioral Neuroscience, 124(5), 559–573. 10.1037/a0020893 20939657 PMC3071152

[IMAG.a.1306-b24] Cabeza, R., Albert, M., Belleville, S., Craik, F. I. M., Duarte, A., Grady, C. L., Lindenberger, U., Nyberg, L., Park, D. C., Reuter-Lorenz, P. A., Rugg, M. D., Steffener, J., & Rajah, M. N. (2018). Maintenance, reserve and compensation: The cognitive neuroscience of healthy ageing. Nature Reviews Neuroscience, 19(11), 701–710. 10.1038/s41583-018-0068-2 PMC6472256 30305711

[IMAG.a.1306-b25] Callow, D. D., Pena, G. S., Stark, C. E. L., & Smith, J. C. (2023). Effects of acute aerobic exercise on mnemonic discrimination performance in older adults. Journal of the International Neuropsychological Society, 29(6), 519–528. 10.1017/S1355617722000492 35968853 PMC10538177

[IMAG.a.1306-b26] Chalmers, R. P., & Flora, D. B. (2015). faoutlier: An R Package for detecting influential cases in exploratory and confirmatory factor analysis. Applied Psychological Measurement, 39(7), 573–574. 10.1177/0146621615597894 29881028 PMC5978519

[IMAG.a.1306-b27] Cheung, G. W., & Rensvold, R. B. (1999). Testing factorial invariance across groups: A reconceptualization and proposed new method. Journal of Management, 25(1), 1–27. 10.1177/014920639902500101

[IMAG.a.1306-b28] Cui, X., Gui, W., Miao, J., Liu, X., Zhu, X., Zheng, Z., Wan, W., Shao, Q., Kray, J., Jiang, Y., & Li, J. (2023). A combined intervention of aerobic exercise and video game in older adults: The efficacy and neural basis on improving mnemonic discrimination. The Journals of Gerontology: Series A, 78(8), 1436–1444. 10.1093/gerona/glac232 36462181

[IMAG.a.1306-b29] Daugherty, A. M., Bender, A. R., Raz, N., & Ofen, N. (2016). Age differences in hippocampal subfield volumes from childhood to late adulthood: Lifespan hippocampal subfield volumes. Hippocampus, 26(2), 220–228. 10.1002/hipo.22517 26286891 PMC4718822

[IMAG.a.1306-b30] de Flores, R., La Joie, R., Landeau, B., Perrotin, A., Mézenge, F., De La Sayette, V., Eustache, F., Desgranges, B., & Chételat, G. (2015). Effects of age and Alzheimer’s disease on hippocampal subfields: Comparison between manual and freesurfer volumetry. Human Brain Mapping, 36(2), 463–474. 10.1002/hbm.22640 25231681 PMC6869780

[IMAG.a.1306-b31] Decker, A. L., & Duncan, K. (2020). Acetylcholine and the complex interdependence of memory and attention. Current Opinion in Behavioral Sciences, 32, 21–28. 10.1016/j.cobeha.2020.01.013

[IMAG.a.1306-b32] Dickhuth, H.-H., & Badtke, G. (Eds.). (2010). Sportmedizin für Ärzte: Lehrbuch auf der Grundlage des Weiterbildungssystems der Deutschen Gesellschaft für Sportmedizin und Prävention (DGSP) (2nd ed.). Deutscher Ärzteverlag. 10.47420/9783769136111

[IMAG.a.1306-b33] Dinoff, A., Herrmann, N., Swardfager, W., & Lanctôt, K. L. (2017). The effect of acute exercise on blood concentrations of brain-derived neurotrophic factor in healthy adults: A meta-analysis. European Journal of Neuroscience, 46(1), 1635–1646. 10.1111/ejn.13603 28493624

[IMAG.a.1306-b34] Düzel, E., Van Praag, H., & Sendtner, M. (2016). Can physical exercise in old age improve memory and hippocampal function? Brain, 139(3), 662–673. 10.1093/brain/awv407 26912638 PMC4766381

[IMAG.a.1306-b35] Eldridge, L. L., Engel, S. A., Zeineh, M. M., Bookheimer, S. Y., & Knowlton, B. J. (2005). A dissociation of encoding and retrieval processes in the human hippocampus. The Journal of Neuroscience, 25(13), 3280–3286. 10.1523/JNEUROSCI.3420-04.2005 15800182 PMC6724896

[IMAG.a.1306-b36] Erickson, K. I., Voss, M. W., Prakash, R. S., Basak, C., Szabo, A., Chaddock, L., Kim, J. S., Heo, S., Alves, H., White, S. M., Wojcicki, T. R., Mailey, E., Vieira, V. J., Martin, S. A., Pence, B. D., Woods, J. A., McAuley, E., & Kramer, A. F. (2011). Exercise training increases size of hippocampus and improves memory. Proceedings of the National Academy of Sciences of the United States of America, 108(7), 3017–3022. 10.1073/pnas.1015950108 21282661 PMC3041121

[IMAG.a.1306-b37] Fabre, C., Chamari, K., Mucci, P., MassÃ©-Biron, J., & PrÃ©faut, C. (2002). Improvement of cognitive function by mental and/or individualized aerobic training in healthy elderly subjects. International Journal of Sports Medicine, 23(6), 415–421. 10.1055/s-2002-33735 12215960

[IMAG.a.1306-b38] Fandakova, Y., Sander, M. C., Grandy, T. H., Cabeza, R., Werkle-Bergner, M., & Shing, Y. L. (2018). Age differences in false memory: The importance of retrieval monitoring processes and their modulation by memory quality. Psychology and Aging, 33(1), 119–133. 10.1037/pag0000212 29494183

[IMAG.a.1306-b39] Fissler, P., Küster, O., Schlee, W., & Kolassa, I.-T. (2013). Novelty interventions to enhance broad cognitive abilities and prevent dementia. Progress in Brain Research, 207, 403–434. 10.1016/B978-0-444-63327-9.00017-5 24309264

[IMAG.a.1306-b40] Frodl, T., Strehl, K., Carballedo, A., Tozzi, L., Doyle, M., Amico, F., Gormley, J., Lavelle, G., & O’Keane, V. (2020). Aerobic exercise increases hippocampal subfield volumes in younger adults and prevents volume decline in the elderly. Brain Imaging and Behavior, 14(5), 1577–1587. 10.1007/s11682-019-00088-6 30927200

[IMAG.a.1306-b41] Gabrieli, J. D. E., Brewer, J. B., Desmond, J. E., & Glover, G. H. (1997). Separate neural bases of two fundamental memory processes in the human medial temporal lobe. Science, 276(5310), 264–266. 10.1126/science.276.5310.264 9092477

[IMAG.a.1306-b42] Güsten, J., Ziegler, G., Düzel, E., & Berron, D. (2021). Age impairs mnemonic discrimination of objects more than scenes: A web-based, large-scale approach across the lifespan. Cortex, 137, 138–148. 10.1016/j.cortex.2020.12.017 33611227

[IMAG.a.1306-b43] Håkansson, K., Ledreux, A., Daffner, K., Terjestam, Y., Bergman, P., Carlsson, R., Kivipelto, M., Winblad, B., Granholm, A.-C., & Mohammed, A. K. H. (2016). BDNF responses in healthy older persons to 35 minutes of physical exercise, cognitive training, and mindfulness: Associations with working memory function. Journal of Alzheimer’s Disease, 55(2), 645–657. 10.3233/JAD-160593 PMC6135088 27716670

[IMAG.a.1306-b44] Hötting, K., & Röder, B. (2013). Beneficial effects of physical exercise on neuroplasticity and cognition. Neuroscience & Biobehavioral Reviews, 37(9), 2243–2257. 10.1016/j.neubiorev.2013.04.005 23623982

[IMAG.a.1306-b45] Hunter, M. D. (2018). State space modeling in an open source, modular, structural equation modeling environment. Structural Equation Modeling: A Multidisciplinary Journal, 25(2), 307–324. 10.1080/10705511.2017.1369354

[IMAG.a.1306-b46] Jaeger, J. (2018). Digit symbol substitution test: The case for sensitivity over specificity in neuropsychological testing. Journal of Clinical Psychopharmacology, 38(5), 513–519. 10.1097/JCP.0000000000000941 30124583 PMC6291255

[IMAG.a.1306-b47] Jutten, R. J., Grandoit, E., Foldi, N. S., Sikkes, S. A. M., Jones, R. N., Choi, S., Lamar, M. L., Louden, D. K. N., Rich, J., Tommet, D., Crane, P. K., & Rabin, L. A. (2020). Lower practice effects as a marker of cognitive performance and dementia risk: A literature review. Alzheimer’s & Dementia: Diagnosis, Assessment & Disease Monitoring, 12(1), e12055. 10.1002/dad2.12055 PMC7346865 32671181

[IMAG.a.1306-b48] Kelly, M. E., Loughrey, D., Lawlor, B. A., Robertson, I. H., Walsh, C., & Brennan, S. (2014). The impact of exercise on the cognitive functioning of healthy older adults: A systematic review and meta-analysis. Ageing Research Reviews, 16, 12–31. 10.1016/j.arr.2014.05.002 24862109

[IMAG.a.1306-b49] Kempermann, G., Fabel, K., Ehninger, D., Babu, H., Leal-Galicia, P., Garthe, A., & Wolf, S. A. (2010). Why and how physical activity promotes experience-induced brain plasticity. Frontiers in Neuroscience, 4, 189. 10.3389/fnins.2010.00189 21151782 PMC3000002

[IMAG.a.1306-b50] Kern, K. L., Storer, T. W., & Schon, K. (2021). Cardiorespiratory fitness, hippocampal subfield volumes, and mnemonic discrimination task performance in aging. Human Brain Mapping, 42(4), 871–892. 10.1002/hbm.25259 33325614 PMC7856657

[IMAG.a.1306-b51] Kievit, R. A., Brandmaier, A. M., Ziegler, G., van Harmelen, A.-L., de Mooij, S. M. M., Moutoussis, M., Goodyer, I. M., Bullmore, E., Jones, P. B., Fonagy, P., Lindenberger, U., & Dolan, R. J. (2018). Developmental cognitive neuroscience using latent change score models: A tutorial and applications. Developmental Cognitive Neuroscience, 33, 99–117. 10.1016/j.dcn.2017.11.007 29325701 PMC6614039

[IMAG.a.1306-b52] Kline, R. B. (2016). Principles and practice of structural equation modeling (4th ed.). The Guilford Press. 10.1080/10705511.2023.2235083

[IMAG.a.1306-b53] Kraft, E. (2012). Cognitive function, physical activity, and aging: Possible biological links and implications for multimodal interventions. Aging, Neuropsychology, and Cognition, 19(1–2), 248–263. 10.1080/13825585.2011.645010 22313174

[IMAG.a.1306-b54] Lacy, J. W., Yassa, M. A., Stark, S. M., Muftuler, L. T., & Stark, C. E. L. (2011). Distinct pattern separation related transfer functions in human CA3/dentate and CA1 revealed using high-resolution fMRI and variable mnemonic similarity. Learning & Memory, 18(1), 15–18. 10.1101/lm.1971111 21164173 PMC3023966

[IMAG.a.1306-b55] Ledergerber, D., & Moser, E. I. (2017). Memory retrieval: Taking the route via subiculum. Current Biology, 27(22), R1225–R1227. 10.1016/j.cub.2017.09.042 29161563

[IMAG.a.1306-b56] Legault, C., Jennings, J. M., Katula, J. A., Dagenbach, D., Gaussoin, S. A., Sink, K. M., Rapp, S. R., Rejeski, W. J., Shumaker, S. A., Espeland, M. A., & the SHARP-P Study Group. (2011). Designing clinical trials for assessing the effects of cognitive training and physical activity interventions on cognitive outcomes: The Seniors Health and Activity Research Program Pilot (SHARP-P) Study, a randomized controlled trial. BMC Geriatrics, 11(1), 27. 10.1186/1471-2318-11-27 21615936 PMC3126708

[IMAG.a.1306-b57] Li, P., Legault, J., & Litcofsky, K. A. (2014). Neuroplasticity as a function of second language learning: Anatomical changes in the human brain. Cortex, 58, 301–324. 10.1016/j.cortex.2014.05.001 24996640

[IMAG.a.1306-b58] Lindenberger, U. (2014). Human cognitive aging: Corriger la fortune? Science, 346(6209), 572–578. 10.1126/science.1254403 25359964

[IMAG.a.1306-b59] Little, T. D., Cunningham, W. A., Shahar, G., & Widaman, K. F. (2002). To parcel or not to parcel: Exploring the question, weighing the merits. Structural Equation Modeling: A Multidisciplinary Journal, 9(2), 151–173. 10.1207/S15328007SEM0902_1

[IMAG.a.1306-b60] Little, T. D., Rhemtulla, M., Gibson, K., & Schoemann, A. M. (2013). Why the items versus parcels controversy needn’t be one. Psychological Methods, 18(3), 285–300. 10.1037/a0033266 23834418 PMC3909043

[IMAG.a.1306-b61] Lustig, C., Shah, P., Seidler, R., & Reuter-Lorenz, P. A. (2009). Aging, training, and the brain: A review and future directions. Neuropsychology Review, 19(4), 504–522. 10.1007/s11065-009-9119-9 19876740 PMC3005345

[IMAG.a.1306-b62] Maass, A., Düzel, S., Goerke, M., Becke, A., Sobieray, U., Neumann, K., Lövden, M., Lindenberger, U., Bäckman, L., Braun-Dullaeus, R., Ahrens, D., Heinze, H.-J., Müller, N. G., & Düzel, E. (2015). Vascular hippocampal plasticity after aerobic exercise in older adults. Molecular Psychiatry, 20(5), 585–593. 10.1038/mp.2014.114 25311366

[IMAG.a.1306-b63] Mårtensson, J., Eriksson, J., Bodammer, N. C., Lindgren, M., Johansson, M., Nyberg, L., & Lövdén, M. (2012). Growth of language-related brain areas after foreign language learning. NeuroImage, 63(1), 240–244. 10.1016/j.neuroimage.2012.06.043 22750568

[IMAG.a.1306-b64] McArdle, J. J., & Hamagami, F. (2001). Latent difference score structural models for linear dynamic analyses with incomplete longitudinal data. In L. M. Collins & A. G. Sayer (Eds.), New methods for the analysis of change. (pp. 139–175). American Psychological Association. 10.1037/10409-005

[IMAG.a.1306-b65] McArdle, J. J., & Nesselroade, J. R. (1994). Using multivariate data to structure developmental change. In S. H. Cohen and H. W. Reese (Eds.), Life-span developmental psychology (pp. 223–267). Psychology Press. 10.4324/9781315792712-10

[IMAG.a.1306-b66] Morris, J. K., Vidoni, E. D., Johnson, D. K., Van Sciver, A., Mahnken, J. D., Honea, R. A., Wilkins, H. M., Brooks, W. M., Billinger, S. A., Swerdlow, R. H., & Burns, J. M. (2017). Aerobic exercise for Alzheimer’s disease: A randomized controlled pilot trial. PLoS One, 12(2), e0170547. 10.1371/journal.pone.0170547 28187125 PMC5302785

[IMAG.a.1306-b67] Neale, M. C., Hunter, M. D., Pritikin, J. N., Zahery, M., Brick, T. R., Kirkpatrick, R. M., Estabrook, R., Bates, T. C., Maes, H. H., & Boker, S. M. (2016). OpenMx 2.0: Extended Structural Equation and Statistical Modeling [Computer software]. http://link.springer.com/10.1007/s11336-014-9435-8 10.1007/s11336-014-9435-8 PMC4516707 25622929

[IMAG.a.1306-b68] Nilsson, J., Berggren, R., Garzón, B., Lebedev, A. V., & Lövdén, M. (2021). Second language learning in older adults: Effects on brain structure and predictors of learning success. Frontiers in Aging Neuroscience, 13, 666851. 10.3389/fnagi.2021.666851 34149398 PMC8209301

[IMAG.a.1306-b69] Nilsson, J., Ekblom, Ö., Ekblom, M., Lebedev, A., Tarassova, O., Moberg, M., & Lövdén, M. (2020). Acute increases in brain-derived neurotrophic factor in plasma following physical exercise relates to subsequent learning in older adults. Scientific Reports, 10(1), 4395. 10.1038/s41598-020-60124-0 32157099 PMC7064503

[IMAG.a.1306-b70] Nyberg, L., & Lindenberger, U. (2020). Brain maintenance and cognition in old age. In D. Poeppel, G. R. Mangun, & M. S. Gazzaniga (Eds.), The cognitive neurosciences (6th ed., pp. 81–89). The MIT Press. 10.7551/mitpress/11442.003.0011

[IMAG.a.1306-b71] Nyberg, L., Lövdén, M., Riklund, K., Lindenberger, U., & Bäckman, L. (2012). Memory aging and brain maintenance. Trends in Cognitive Sciences, 16(5), 292–305. 10.1016/j.tics.2012.04.005 22542563

[IMAG.a.1306-b72] Nyberg, L., & Pudas, S. (2019). Successful memory aging. Annual Review of Psychology, 70(1), 219–243. 10.1146/annurev-psych-010418-103052 29949727

[IMAG.a.1306-b73] Oswald, W. D., Gunzelmann, T., Rupprecht, R., & Hagen, B. (2006). Differential effects of single versus combined cognitive and physical training with older adults: The SimA study in a 5-year perspective. European Journal of Ageing, 3(4), 179–192. 10.1007/s10433-006-0035-z 28794762 PMC5546372

[IMAG.a.1306-b74] Posit Team. (2023). RStudio: Integrated Development Environment for R (Version 2023.06.2+561) [Computer software]. Posit Software, PBC. http://www.posit.co/

[IMAG.a.1306-b75] Pritikin, J. N., Hunter, M. D., & Boker, S. M. (2015). Modular open-source software for item factor analysis. Educational and Psychological Measurement, 75(3), 458–474. 10.1177/0013164414554615 27065479 PMC4822086

[IMAG.a.1306-b76] R Core Team. (2023). R: A Language and Environment for Statistical Computing [Computer software]. R Foundation for Statistical Computing. https://www.R-project.org/

[IMAG.a.1306-b77] Raz, N., Lindenberger, U., Rodrigue, K. M., Kennedy, K. M., Head, D., Williamson, A., Dahle, C., Gerstorf, D., & Acker, J. D. (2005). Regional brain changes in aging healthy adults: General trends, individual differences and modifiers. Cerebral Cortex, 15(11), 1676–1689. 10.1093/cercor/bhi044 15703252

[IMAG.a.1306-b78] Rönnlund, M., Nyberg, L., Bäckman, L., & Nilsson, L.-G. (2005). Stability, growth, and decline in adult life span development of declarative memory: Cross-sectional and longitudinal data from a population-based study. Psychology and Aging, 20(1), 3–18. 10.1037/0882-7974.20.1.3 15769210

[IMAG.a.1306-b79] Rosano, C., Guralnik, J., Pahor, M., Glynn, N. W., Newman, A. B., Ibrahim, T. S., Erickson, K., Cohen, R., Shaaban, C. E., MacCloud, R. L., & Aizenstein, H. J. (2017). Hippocampal response to a 24-month physical activity intervention in sedentary older adults. The American Journal of Geriatric Psychiatry, 25(3), 209–217. 10.1016/j.jagp.2016.11.007 27986412 PMC5568026

[IMAG.a.1306-b80] Roy, D. S., Kitamura, T., Okuyama, T., Ogawa, S. K., Sun, C., Obata, Y., Yoshiki, A., & Tonegawa, S. (2017). Distinct neural circuits for the formation and retrieval of episodic memories. Cell, 170(5), 1000.e19–1012.e19. 10.1016/j.cell.2017.07.013 28823555 PMC5586038

[IMAG.a.1306-b81] Schaie, K. W. (1994). The course of adult intellectual development. American Psychologist, 49(4), 304–313. 10.1037/0003-066X.49.4.304 8203802

[IMAG.a.1306-b82] Schermelleh-Engel, K., Moosbrugger, H., & Müller, H. (2003). Evaluating the fit of structural equation models: Tests of significance and descriptive goodness-of-fit measures. Methods of Psychological Research, 8(2), 23–74. 10.1037/met0000680.supp

[IMAG.a.1306-b110] Schulz, K. F., Altman, D. G., & Moher, D. for the CONSORT Group. (2010). CONSORT 2010 Statement: Updated guidelines for reporting parallel group randomised trials. BMJ, 340, c332. 10.1136/bmj.c332 20332509 PMC2844940

[IMAG.a.1306-b83] Shing, Y. L., Rodrigue, K. M., Kennedy, K. M., Fandakova, Y., Bodammer, N., Werkle-Bergner, M., Lindenberger, U., & Raz, N. (2011). Hippocampal subfield volumes: Age, vascular risk, and correlation with associative memory. Frontiers in Aging Neuroscience, 3, 2. 10.3389/fnagi.2011.00002 21331174 PMC3035014

[IMAG.a.1306-b84] Snodgrass, J. G., & Corwin, J. (1988). Pragmatics of measuring recognition memory: Applications to dementia and amnesia. Journal of Experimental Psychology: General, 117(1), 34–50. 10.1037/0096-3445.117.1.34 2966230

[IMAG.a.1306-b85] Stark, S. M., Yassa, M. A., Lacy, J. W., & Stark, C. E. L. (2013). A task to assess behavioral pattern separation (BPS) in humans: Data from healthy aging and mild cognitive impairment. Neuropsychologia, 51(12), 2442–2449. 10.1016/j.neuropsychologia.2012.12.014 23313292 PMC3675184

[IMAG.a.1306-b86] Stark, S. M., Yassa, M. A., & Stark, C. E. L. (2010). Individual differences in spatial pattern separation performance associated with healthy aging in humans. Learning & Memory, 17(6), 284–288. 10.1101/lm.1768110 20495062 PMC2884287

[IMAG.a.1306-b87] Szuhany, K. L., Bugatti, M., & Otto, M. W. (2015). A meta-analytic review of the effects of exercise on brain-derived neurotrophic factor. Journal of Psychiatric Research, 60, 56–64. 10.1016/j.jpsychires.2014.10.003 25455510 PMC4314337

[IMAG.a.1306-b88] Tarassova, O., Ekblom, M. M., Moberg, M., Lövdén, M., & Nilsson, J. (2020). Peripheral BDNF response to physical and cognitive exercise and its association with cardiorespiratory fitness in healthy older adults. Frontiers in Physiology, 11, 1080. 10.3389/fphys.2020.01080 32982796 PMC7477111

[IMAG.a.1306-b89] ten Brinke, L. F., Bolandzadeh, N., Nagamatsu, L. S., Hsu, C. L., Davis, J. C., Miran-Khan, K., & Liu-Ambrose, T. (2015). Aerobic exercise increases hippocampal volume in older women with probable mild cognitive impairment: A 6-month randomised controlled trial. British Journal of Sports Medicine, 49(4), 248–254. 10.1136/bjsports-2013-093184 24711660 PMC4508129

[IMAG.a.1306-b90] Thomas, A. K., & Gutchess, A. (Eds.). (2020). The Cambridge handbook of cognitive aging: A life course perspective (1st ed.). Cambridge University Press. 10.1017/9781108552684

[IMAG.a.1306-b91] Toner, C. K., Pirogovsky, E., Kirwan, C. B., & Gilbert, P. E. (2009). Visual object pattern separation deficits in nondemented older adults. Learning & Memory, 16(5), 338–342. 10.1101/lm.1315109 19403797

[IMAG.a.1306-b92] van Praag, H. (2008). Neurogenesis and exercise: Past and future directions. NeuroMolecular Medicine, 10(2), 128–140. 10.1007/s12017-008-8028-z 18286389

[IMAG.a.1306-b111] Voss, M. W., Soto, C., Yoo, S., Sodoma, M., Vivar, C., & Van Praag, H. (2019). Exercise and Hippocampal Memory Systems. Trends in Cognitive Sciences, 23(4), 318–333. 10.1016/j.tics.2019.01.006 30777641 PMC6422697

[IMAG.a.1306-b93] Walhovd, K. B., Westerhausen, R., de Lange, A.-M. G., Bråthen, A. C. S., Grydeland, H., Engvig, A., & Fjell, A. M. (2016). Premises of plasticity—And the loneliness of the medial temporal lobe. NeuroImage, 131, 48–54. 10.1016/j.neuroimage.2015.10.060 26505299

[IMAG.a.1306-b94] Ware, C., Dautricourt, S., Gonneaud, J., & Chételat, G. (2021). Does second language learning promote neuroplasticity in aging? A systematic review of cognitive and neuroimaging studies. Frontiers in Aging Neuroscience, 13, 706672. 10.3389/fnagi.2021.706672 34867264 PMC8633567

[IMAG.a.1306-b95] Wechsler, D. (1981). The psychometric tradition: Developing the Wechsler adult intelligence scale. Contemporary Educational Psychology, 6(2), 82–85. 10.1016/0361-476X(81)90035-7

[IMAG.a.1306-b96] Wenger, E., Düzel, S., Polk, S. E., Bodammer, N. C., Misgeld, C., Porst, J., Wolfarth, B., Kühn, S., & Lindenberger, U. (2022). Vamos en bici: Study protocol of an investigation of cognitive and neural changes following language training, physical exercise training, or a combination of both. bioRxiv. 10.1101/2022.01.30.478181

[IMAG.a.1306-b112] Wenger, E., Kohncke, Y., Polk, S. E., Duzel, S., Kleemeyer, M. M., Bodammer, N. C., Brandmaier, A. M., Kuhn, S., & Lindenberger, U. (in preparation). Language-learning in older adults induced changes in episodic memory and gray matter volume but no added benefit of combined language and exercise training. Center for Lifespan Psychology, Max Planck Institute for Human Development, Berlin.

[IMAG.a.1306-b97] Whiteman, A. S., Young, D. E., He, X., Chen, T. C., Wagenaar, R. C., Stern, C. E., & Schon, K. (2014). Interaction between serum BDNF and aerobic fitness predicts recognition memory in healthy young adults. Behavioural Brain Research, 259, 302–312. 10.1016/j.bbr.2013.11.023 24269495 PMC3991014

[IMAG.a.1306-b98] Wickham, H., Averick, M., Bryan, J., Chang, W., McGowan, L., François, R., Grolemund, G., Hayes, A., Henry, L., Hester, J., Kuhn, M., Pedersen, T., Miller, E., Bache, S., Müller, K., Ooms, J., Robinson, D., Seidel, D., Spinu, V.,… Yutani, H. (2019). Welcome to the Tidyverse. Journal of Open Source Software, 4(43), 1686. 10.21105/joss.01686

[IMAG.a.1306-b99] Widaman, K. F., Ferrer, E., & Conger, R. D. (2010). Factorial invariance within longitudinal structural equation models: Measuring the same construct across time. Child Development Perspectives, 4(1), 10–18. 10.1111/j.1750-8606.2009.00110.x 20369028 PMC2848495

[IMAG.a.1306-b100] Wilckens, K. A., Stillman, C. M., Waiwood, A. M., Kang, C., Leckie, R. L., Peven, J. C., Foust, J. E., Fraundorf, S. H., & Erickson, K. I. (2021). Exercise interventions preserve hippocampal volume: A meta‐analysis. Hippocampus, 31(3), 335–347. 10.1002/hipo.23292 33315276 PMC11497212

[IMAG.a.1306-b101] Yassa, M. A., Lacy, J. W., Stark, S. M., Albert, M. S., Gallagher, M., & Stark, C. E. L. (2010). Pattern separation deficits associated with increased hippocampal CA3 and dentate gyrus activity in nondemented older adults. Hippocampus, 21(9), 968–979. 10.1002/hipo.20808 20865732 PMC3010452

[IMAG.a.1306-b102] Yeung, L.-K., Ryan, J. D., Cowell, R. A., & Barense, M. D. (2013). Recognition memory impairments caused by false recognition of novel objects. Journal of Experimental Psychology: General, 142(4), 1384–1397. 10.1037/a0034021 23937183

[IMAG.a.1306-b103] Young, J., Angevaren, M., Rusted, J., & Tabet, N. (2015). Aerobic exercise to improve cognitive function in older people without known cognitive impairment. Cochrane Database of Systematic Reviews, 2015(4), CD005381. 10.1002/14651858.CD005381.pub4 25900537 PMC10554155

[IMAG.a.1306-b104] Zammit, A. R., Ezzati, A., Zimmerman, M. E., Lipton, R. B., Lipton, M. L., & Katz, M. J. (2017). Roles of hippocampal subfields in verbal and visual episodic memory. Behavioural Brain Research, 317, 157–162. 10.1016/j.bbr.2016.09.038 27646772 PMC6343125

[IMAG.a.1306-b105] Zeineh, M. M., Engel, S. A., Thompson, P. M., & Bookheimer, S. Y. (2003). Dynamics of the hippocampus during encoding and retrieval of face-name pairs. Science, 299(5606), 577–580. 10.1126/science.1077775 12543980

[IMAG.a.1306-b106] Zhang, L., Mak, E., Reilhac, A., Shim, H. Y., Ng, K. K., Ong, M. Q. W., Ji, F., Chong, E. J. Y., Xu, X., Wong, Z. X., Stephenson, M. C., Venketasubramanian, N., Tan, B. Y., O’Brien, J. T., Zhou, J. H., Chen, C. L. H., & the Alzheimer’s Disease Neuroimaging Initiative. (2020). Longitudinal trajectory of Amyloid‐related hippocampal subfield atrophy in nondemented elderly. Human Brain Mapping, 41(8), 2037–2047. 10.1002/hbm.24928 31944479 PMC7267893

[IMAG.a.1306-b107] Zong, B., Yu, F., Zhang, X., Zhao, W., Sun, P., Li, S., & Li, L. (2022). Understanding how physical exercise improves Alzheimer’s disease: Cholinergic and monoaminergic systems. Frontiers in Aging Neuroscience, 14, 869507. 10.3389/fnagi.2022.869507 35663578 PMC9158463

