# Supplementary material for: Beneficial effects of foreign language learning and aerobic exercise on dentate gyrus volume and mnemonic discrimination in healthy older adults: Results from a randomized controlled trial
Source: Imaging Neurosci (Camb). 2026 Jul 24;4:IMAG.a.1306. doi: 10.1162/IMAG.a.1306 (PMC13403954; doi:10.1162/IMAG.a.1306)
Supplement: Supplementary Material [file IMAG.a.1306_supp.pdf]

Supplementary Material for

Beneficial effects of foreign language learning and aerobic exercise on dentate gyrus volume and mnemonic discrimination in healthy older adults: Results from a randomized controlled trial

Polk et al., 2026

Supplementary Tables 4
Supplementary Table S1 | *Consolidated Standards of Reporting Trials (CONSORT) 2010 Checklist according to Schulz et al. (2010).* 4
Supplementary Table S2 | *Means and standard deviations of indicator variables at T1 comparing compliant and non-compliant participants.* 7
Supplementary Table S3 | *Means and standard deviations of hours spent training per group, as well as pairwise group comparisons of time spent training.* 8
Supplementary Table S4 | *Means and standard deviations of all indicator variables, as well as group difference statistics calculated with ANOVAs.* 9
Supplementary Table S5 | *Covariance of indicator variables at T1*. 10
Supplementary Table S6 | *Model performance metrics.* 11
Supplementary Table S7 | Model parameters: Latent change score model of cornu ammonis 1/2 volume. 12
Supplementary Table S8 | *Model parameters: Latent change score model of cornu ammonis 3/dentate gyrus volume.* 13
Supplementary Table S9 | *Model parameters: Latent change score model of subiculum volume.* 14
Supplementary Table S10 | Model parameters: Latent change score model of entorhinal cortex volume. 15
Supplementary Table S11 | *Model parameters: Latent change score model of Mnemonic Discrimination Task for Objects and Scenes performance.* 16
Supplementary Table S12 | Model parameters of interest: bivariate associations between hippocampal subfield volume and Mnemonic Discrimination Task for Objects and Scenes performance. 17
Supplementary Figures 18
Supplementary Figure S1 | Univariate (boxplot) and multivariate (spaghetti plot) outliers (blue). Univariate outliers were defined as those falling > 4 SDs away from the mean. Multivariate outliers were determined using the classical product-moment method (criterion = .001). 18
Supplementary Figure S2 | Distribution of manifest variables at each time point (violin) and individual change trajectories (spaghetti), as well as the mean (black dot) and standard deviation (error bars) at each time point and for each group. 19
Supplementary Figure S3 | Bivariate latent change score models (LCSMs) including (a) covariates of no interest (age, sex, years of education) or (b) change in cardiovascular fitness as a pseudo-LCSM. 20
Supplementary Methods 21
Measuring physical fitness 21
Measuring baseline cognitive function 21
Imaging data acquisition and preprocessing 21
Measuring mnemonic discrimination 22

Multivariate latent change score models.. 23
Supplementary Results 25
Regarding the interpretation of model estimates 25
Regarding baseline differences in hippocampal subfield volume. 25
Sensitivity analyses: outliers and compliance 25
Brain-derived neurotrophic factor does not predict mnemonic discrimination 26
Supplementary References 27

Supplementary Tables

Supplementary Table S1 | *Consolidated Standards of Reporting Trials (CONSORT) 2010 Checklist according to Schulz et al. (2010).*

Section/Topic	Item No	Checklist item	Reported on page # / Details
**Title and abstract**			
	1a	Identification as a randomized trial in the title	p. 1
	1b	Structured summary of trial design, methods, results, and conclusions (for specific guidance see CONSORT for abstracts)	p. 2
**Introduction**			
Background and objectives	2a	Scientific background and explanation of rationale	pp. 3–5
	2b	Specific objectives or hypotheses	pp. 5–6
**Methods**			
Trial design	3a	Description of trial design (such as parallel, factorial) including allocation ratio	Randomized parallel group design with four groups and equal allocation.
	3b	Important changes to methods after trial commencement (such as eligibility criteria), with reasons	A compliance threshold of 1890 total minutes was set *post hoc*.
Participants	4a	Eligibility criteria for participants	p. 7
	4b	Settings and locations where the data were collected	p. 7
Interventions	5	The interventions for each group with sufficient details to allow replication, including how and when they were actually administered	pp. 9–10
Outcomes	6a	Completely defined pre-specified primary and secondary outcome measures, including how and when they were assessed	pp. 11–13
	6b	Any changes to trial outcomes after the trial commenced, with reasons	n/a
Sample size	7a	How sample size was determined	A power analysis was conducted with a previous unpublished version of semPower (Moshagen & Bader, 2024, *Behavior Research Methods*).
	7b	When applicable, explanation of any interim analyses and stopping guidelines	n/a
Randomization:			
Sequence generation	8a	Method used to generate the random allocation sequence	p. 7
	8b	Type of randomization; details of any restriction (such as blocking and block size)	p. 7
Allocation concealment mechanism	9	Mechanism used to implement the random allocation sequence (such as sequentially numbered containers), describing any steps	Assignment to groups was done by a separate recruitment team who were not involved in the data analyses.

		taken to conceal the sequence until interventions were assigned	
Implementation	10	Who generated the random allocation sequence, who enrolled participants, and who assigned participants to interventions	Assignment to groups was done by a separate recruitment team who were not involved in the data analyses.
Blinding	11a	If done, who was blinded after assignment to interventions (for example, participants, care providers, those assessing outcomes) and how	p. 7
	11b	If relevant, description of the similarity of interventions	pp. 9–10
Statistical methods	12a	Statistical methods used to compare groups for primary and secondary outcomes	pp. 13–14
	12b	Methods for additional analyses, such as subgroup analyses and adjusted analyses	pp. 13–14
**Results**			
Participant flow	13a	For each group, the numbers of participants who were randomly assigned, received intended treatment, and were analyzed for the primary outcome	pp. 7–9 and Figure 1
	13b	For each group, losses and exclusions after randomization, together with reasons	pp. 7–9 and Figure 1
Recruitment	14a	Dates defining the periods of recruitment and follow-up	pp. 7–8
	14b	Why the trial ended or was stopped	The predetermined sample size was attained. The trial itself was planned to run for six months.
Baseline data	15	A table showing baseline demographic and clinical characteristics for each group	Table 1
Numbers analyzed	16	For each group, number of participants (denominator) included in each analysis and whether the analysis was by original assigned groups	p. 9 and Table 1
Outcomes and estimation	17a	For each primary and secondary outcome, results for each group, and the estimated effect size and its precision (such as 95% confidence interval)	pp. 19–22 and Supplementary Tables
	17b	For binary outcomes, presentation of both absolute and relative effect sizes is recommended	n/a
Ancillary analyses	18	Results of any other analyses performed, including subgroup analyses and adjusted analyses, distinguishing pre-specified from exploratory	pp. 23–24: Sensitivity analyses including age, sex, and education in the models and a *post hoc* analysis of the influence VO_2peak as a measure of cardiovascular fitness on the results.

Harms	19	All important harms or unintended effects in each group (for specific guidance see CONSORT for harms)	The interventions in the current trial comprised lifestyle changes (i.e., language learning and/or exercise), which are not typically associated with adverse events. Participant in the active control group was not assumed to be associated with any harms (i.e., withholding of treatment) for this group of healthy older adults. Discomfort in the MRI was quantified with a post-measurement questionnaire and participants were clearly informed that their participation was voluntary, and they could stop the trial at any point.
**Discussion**			
Limitations	20	Trial limitations, addressing sources of potential bias, imprecision, and, if relevant, multiplicity of analyses	p. 33
Generalizability	21	Generalizability (external validity, applicability) of the trial findings	p. 33
Interpretation	22	Interpretation consistent with results, balancing benefits and harms, and considering other relevant evidence	pp. 25–32
**Other information**			
Registration	23	Registration number and name of trial registry	n/a
Protocol	24	Where the full trial protocol can be accessed, if available	p. 7: Wenger, E., Düzel, S., Polk, S. E., Bodammer, N. C., Misgeld, C., Porst, J., Wolfarth, B., Kühn, S., & Lindenberger, U. (2022). *Vamos en bici: Study protocol of an investigation of cognitive and neural changes following language training, physical exercise training, or a combination of both* [Preprint]. bioRxiv. https://doi.org/10.1101/2022.01.30.478181
Funding	25	Sources of funding and other support (such as supply of drugs), role of funders	p. 35

Supplementary Table S2 | *Means and standard deviations of indicator variables at T1 comparing compliant and non-compliant participants.*

Variable	Compliant (n = 126)	Non-compliant (n = 16)	F statistic and effect size η^2 of group difference
Left CA1/CA2 volume	192 ± 30.1	191 ± 27.9	$F_{1,137} = 0.01$, $\eta^2 = 0.000$
Right CA1/CA2 volume	205 ± 32.3	191 ± 32.3	$F_{1,138} = 2.54$, $\eta^2 = 0.018$
Left CA3/DG volume	256 ± 50.2	265 ± 46.3	$F_{1,138} = 0.43$, $\eta^2 = 0.003$
Right CA3/DG volume	245 ± 42.8	234 ± 36.7	$F_{1,136} = 0.99$, $\eta^2 = 0.007$
Left subiculum volume	336 ± 60.0	328 ± 43.3	$F_{1,138} = 0.23$, $\eta^2 = 0.002$
Right subiculum volume	311 ± 54.4	290 ± 39.2	$F_{1,137} = 1.96$, $\eta^2 = 0.014$
Left ERC volume	240 ± 47.3	235 ± 54.3	$F_{1,136} = 0.15$, $\eta^2 = 0.001$
Right ERC volume	218 ± 42.5	229 ± 34.6	$F_{1,138} = 0.87$, $\eta^2 = 0.006$
MDT parcel 1 (CHR)	0.27 ± 0.198	0.20 ± 0.182	$F_{1,130} = 1.42$, $\eta^2 = 0.011$
MDT parcel 2 (CHR)	0.27 ± 0.205	0.28 ± 0.154	$F_{1,130} = 0.03$, $\eta^2 = 0.000$
MDT Parcel 3 (CHR)	0.28 ± 0.210	0.27 ± 0.190	$F_{1,130} = 0.02$, $\eta^2 = 0.000$
VO_2peak	22.8 ± 5.36	20.4 ± 4.63	$F_{1,136} = 2.32$, $\eta^2 = 0.017$

Note. CA = *cornu ammonis*, DG = dentate gyrus, ERC = entorhinal cortex, MDT = Mnemonic Discrimination Task, CHR = corrected hit rate.

Supplementary Table S3 | *Means and standard deviations of hours spent training per group, as well as pairwise group comparisons of time spent training.*

		Active control group (ACG)	Language group (LG)	Exercise group (EG)	Language + exercise group (L+EG)
Total time spent training	ACG	M = 80.3, SD = 30.2			
	LG	t_{59} = 1.35, *p* = .182	M = 92.1, SD = 38.2		
	EG	t_{68} = 3.86, *p* < .001	t_{65} = 1.82, *p* = .073	M = 106.6, SD = 26.9	
	L+EG	t_{57} = 0.77, *p* = .446	t_{54} = −0.57, *p* = .573	t_{63} = −2.66, *p* = .010	M = 86.7, SD = 33.7
Time spent reading	ACG	M = 80.3, SD = 30.2			
	LG	t_{59} = −6.91, *p* < .001	M = 32.4, SD = 22.9		
	EG	t_{68} = −4.10, *p* < .001	t_{65} = 4.20, *p* < .001	M = 55.1, SD = 21.0	
Time spent learning Spanish	LG		M = 59.7, SD = 22.4		
	L+EG		t_{54} = −2.16, *p* = .035		M = 45.5, SD = 26.9
Time spent exercising	EG			M = 51.6, SD = 12.4	
	L+EG			t_{63} = −3.20, *p* = .002	M = 41.2, SD = 13.5
Time spent learning Spanish and/or exercising	LG		M = 59.7, SD = 22.4		
	EG		t_{65} = −1.89, *p* = .063	M = 51.6, SD = 12.4	
	L+EG		t_{54} = 3.55, *p* < .001	t_{63} = 5.90, *p* < .001	M = 86.7, SD = 33.7

Note. Means (M) and standard deviations (SD) are shown on the diagonal, and pairwise *t*-tests with uncorrected *p*-values are shown in the lower triangle.

Supplementary Table S4 | *Means and standard deviations of all indicator variables, as well as group difference statistics calculated with ANOVAs.*

Variable	Time point	ACG	LG	EG	L+EG	Group difference
**Hippocampal subfield volume (mm³)**						
Left CA1/CA2	T1	191 ± 29.1	184 ± 28.6	194 ± 31.4	198 ± 28.9	$F_{1,137}$ = 1.64, p = .202
Right CA1/CA2	T1	204 ± 33.2	203 ± 32.2	202 ± 35.9	205 ± 28.5	$F_{1,138}$ = 0.02, p = .887
Left CA1/CA2	T2	189 ± 29.4	185 ± 27.6	195 ± 30.8	194 ± 29.9	$F_{1,122}$ = 1.07, p = .303
Right CA1/CA2	T2	204 ± 35.9	201 ± 30.0	205 ± 34.7	209 ± 34.8	$F_{1,123}$ = 0.40, p = .530
Left CA1/CA2	T3	189 ± 35.0	186 ± 32.1	190 ± 29.5	192 ± 37.8	$F_{1,122}$ = 0.18, p = .675
Right CA1/CA2	T3	200 ± 33.5	206 ± 31.0	202 ± 27.3	206 ± 38.5	$F_{1,123}$ = 0.32, p = .575
Left CA3/DG	T1	248 ± 43.7	249 ± 48.1	263 ± 54.4	269 ± 50.1	**$F_{1,138}$ = 4.12, p = .044**
Right CA3/DG	T1	238 ± 38.7	244 ± 42.4	244 ± 50.5	249 ± 35.1	$F_{1,136}$ = 1.04, p = .309
Left CA3/DG	T2	247 ± 49.6	255 ± 51.1	260 ± 52.9	264 ± 46.9	$F_{1,123}$ = 1.86, p = .175
Right CA3/DG	T2	238 ± 32.6	246 ± 41.2	250 ± 50.2	250 ± 35.7	$F_{1,121}$ = 1.51, p = .221
Left CA3/DG	T3	243 ± 47.5	259 ± 52.9	250 ± 48.5	264 ± 50.1	$F_{1,123}$ = 1.50, p = .223
Right CA3/DG	T3	230 ± 40.5	246 ± 35.5	246 ± 48.7	250 ± 48.1	$F_{1,121}$ = 2.79, p = .097
Left subiculum	T1	329 ± 63.4	318 ± 58.2	346 ± 62.6	344 ± 42.1	$F_{1,138}$ = 2.78, p = .098
Right subiculum	T1	303 ± 57.3	302 ± 48.8	319 ± 53.3	301 ± 46.8	$F_{1,137}$ = 0.15, p = .698
Left subiculum	T2	321 ± 63.8	328 ± 68.5	342 ± 71.7	333 ± 53.0	$F_{1,123}$ = 1.14, p = .288
Right subiculum	T2	303 ± 51.6	307 ± 52.4	327 ± 60.8	315 ± 47.5	$F_{1,122}$ = 1.81, p = .181
Left subiculum	T3	318 ± 62.0	328 ± 65.8	328 ± 61.7	340 ± 52.0	$F_{1,123}$ = 1.58, p = .211
Right subiculum	T3	297 ± 56.0	306 ± 50.2	323 ± 63.2	310 ± 51.9	$F_{1,122}$ = 1.78, p = .185
Left ERC	T1	228 ± 42.0	226 ± 45.5	258 ± 51.0	242 ± 46.9	**$F_{1,136}$ = 4.14, p = .044**
Right ERC	T1	218 ± 37.8	220 ± 43.3	219 ± 41.8	221 ± 45.9	$F_{1,138}$ = 0.06, p = .813
Left ERC	T2	231 ± 42.4	226 ± 42.2	256 ± 51.2	239 ± 42.1	$F_{1,121}$ = 2.60, p = .110
Right ERC	T2	220 ± 31.4	212 ± 47.6	219 ± 40.9	222 ± 38.6	$F_{1,123}$ = 0.11, p = .746
Left ERC	T3	226 ± 50.7	227 ± 54.0	258 ± 55.4	246 ± 47.0	**$F_{1,121}$ = 4.84, p = .030**
Right ERC	T3	216 ± 40.7	211 ± 46.6	217 ± 40.4	218 ± 43.1	$F_{1,123}$ = 0.14, p = .713
**MDT-OS performance (corrected hit rate)**						
Parcel 1	T1	0.29 ± 0.214	0.28 ± 0.185	0.21 ± 0.188	0.26 ± 0.198	$F_{1,130}$ = 1.30, p = .256
Parcel 2	T1	0.27 ± 0.196	0.26 ± 0.193	0.25 ± 0.199	0.29 ± 0.217	$F_{1,130}$ = 0.05, p = .827
Parcel 3	T1	0.29 ± 0.181	0.27 ± 0.204	0.27 ± 0.226	0.27 ± 0.225	$F_{1,130}$ = 0.12, p = .724
Parcel 1	T2	0.27 ± 0.229	0.27 ± 0.242	0.24 ± 0.169	0.28 ± 0.200	$F_{1,117}$ = 0.00, p = .946
Parcel 2	T2	0.30 ± 0.217	0.25 ± 0.145	0.26 ± 0.197	0.33 ± 0.161	$F_{1,117}$ = 0.26, p = .612
Parcel 3	T2	0.32 ± 0.185	0.25 ± 0.184	0.30 ± 0.181	0.32 ± 0.165	$F_{1,117}$ = 0.02, p = .901
Parcel 1	T3	0.29 ± 0.171	0.30 ± 0.213	0.32 ± 0.141	0.30 ± 0.165	$F_{1,118}$ = 0.14, p = .706
Parcel 2	T3	0.25 ± 0.162	0.27 ± 0.192	0.26 ± 0.190	0.27 ± 0.165	$F_{1,118}$ = 0.18, p = .675
Parcel 3	T3	0.30 ± 0.202	0.27 ± 0.225	0.30 ± 0.189	0.36 ± 0.195	$F_{1,118}$ = 1.40, p = .239
**Peak oxygen uptake (mL/min/kg)**						
VO_2peak	T1	22.3 ± 6.36	21.3 ± 3.87	23.8 ± 5.64	22.1 ± 4.41	$F_{1,136}$ = 0.23, p = .634
VO_2peak	T3	24.2 ± 6.58	21.8 ± 4.58	26.0 ± 5.85	24.5 ± 4.84	$F_{1,118}$ = 1.40, p = .240

Note. CA = *cornu ammonis*, DG = dentate gyrus, ERC = entorhinal cortex, MDT-OS = Mnemonic Discrimination Task for Objects and Scenes, T1 = time point 1 (0 months), T2 = time point 2 (3 months), T3 = time point three (6 months), ACG = active control group, LG = language group, EG = exercise group, L+EG = language + exercise group. Group difference statistics in bold are significant at p < .050.

Supplementary Table S5 | *Covariance of indicator variables at T1.*

	1	2	3	4	5	6	7	8	9	10	11	12	13	14
1. Left CA1/CA2 (mm^3)	—													
2. Right CA1/CA2 (mm^3)	.59*	—												
3. Left CA3/DG (mm^3)	.68*	.46*	—											
4. Right CA3/DG (mm^3)	.40*	.69*	.58*	—										
5. Left subiculum (mm^3)	.58*	.31*	.57*	.34*	—									
6. Right subiculum (mm^3)	.40*	.50*	.41*	.61*	.57*	—								
7. Left ERC (mm^3)	.38*	.28*	.24*	.26*	.39*	.29*	—							
8. Right ERC (mm^3)	.27*	.32*	.18*	.32*	.19*	.31*	.52*	—						
9. MDT parcel 1 (CHR)	.00	.07	−.01	.09	−.08	−.03	−.09	−.03	—					
10. MDT parcel 2 (CHR)	.06	.06	−.03	.13	−.02	.05	−.03	−.05	.42*	—				
11. MDT Parcel 3 (CHR)	.07	.06	.08	.10	−.01	.02	−.12	−.02	.55*	.47*	—			
12. VO_2peak (mL/min/kg)	−.04	−.01	.01	.02	−.02	.01	.04	.11	−.08	.04	.00	—		
13. Age (years)	−.20*	−.25*	−.02	−.21*	−.10	−.27*	−.13	−.25*	−.21*	−.10	−.21*	−.10	—	
14. Sex	.04	.04	−.02	−.04	.06	.13	.03	.06	−.09	−.12	−.11	−.40*	−.10	—
15. Education (years)	.03	−.02	.03	.02	.01	−.10	−.06	−.10	.16	.15	.02	.13	.06	−.28*

Note. CA = *cornu ammonis*, DG = dentate gyrus, ERC = entorhinal cortex, MDT = Mnemonic Discrimination Task, CHR = corrected hit rate. Sex coded as 1 = male, 2 = female.
*Significant correlation at $p < .050$, uncorrected.

Supplementary Table S6 | *Model performance metrics.*

	Group invariance	Time invariance	Fit statistics		Variance in change
	None vs. strict	None vs. strict			
Model	χ^2_6	χ^2_4	RMSEA [95% CI]	CFI	*Z*-score
CA1/CA2 volume	1.11	4.27	.026 [.000, .091]	.996	4.142**
CA3/DG volume	6.43	16.53*	.000 [.000, .066]	1.000	5.009**
Subiculum volume	2.53	1.30	.000 [.000, .075]	1.000	3.972**
ERC volume	1.27	4.76	.015 [.000, .086]	.999	3.299**

	None vs. metric	Metric vs. strict	None vs. metric	Metric vs. strict	Fit statistics		Variance in change
	χ^2_6	χ^2_3	χ^2_4	χ^2_2	RMSEA [95% CI]	CFI	*Z*-score
MDT-OS	5.67	2.62	3.33	3.02	.016 [.000, .065]	.994	2.590**

	Fit statistics	
	RMSEA [95% CI]	CFI
CA1/CA2 volume ~ MDT-OS	.033 [.000, .060]	.976
CA3/DG volume ~ MDT-OS	.023 [.000, .054]	.989
Subiculum volume ~ MDT-OS	.000 [.000, .043]	1.000
ERC volume ~ MDT-OS	.029 [.000, .057]	.988

Note. CA = *cornu ammonis*, DG = dentate gyrus, ERC = entorhinal cortex, MDT-OS = Mnemonic Discrimination Task for Objects and Scenes, RMSEA = root mean square error of approximation, CI = confidence intervals, CFI = comparative fit index.
* $p < .050$ indicating significant factorial variance; ** $p < .050$ indicating significant variance in change.

Supplementary Table S7 | Model parameters: Latent change score model of cornu ammonis 1/2 volume.

Parameters: across all groups	Unstandardized estimate (*b*) [95% CI]	Standard error	*Z*-score	*p*-value
RES_{left}	0.448 [0.391, 0.505]	0.029	15.448	.000
RES_{right}	0.443 [0.382, 0.504]	0.031	14.290	.000
COV_{hemi}	0.209 [0.159, 0.259]	0.025	8.360	.000
VAR_{HC1}	0.550 [0.405, 0.695]	0.074	7.432	.000
$VAR_{\Delta HC}$	0.139 [0.073, 0.205]	0.033	4.212	.000
$COV_{HC1\sim\Delta HC12}$	−0.079 [−0.162, 0.004]	0.042	−1.881	.060
$COV_{HC1\sim\Delta HC23}$	−0.012 [−0.079, 0.055]	0.034	−0.353	.724
$COV_{\Delta HC12\sim\Delta HC23}$	−0.040 [−0.098, 0.018]	0.030	−1.333	.183
M_{HC1}	0.030 [−0.083, 0.143]	0.058	0.517	.605
$M_{\Delta HC}$ (Main effect of time)	−0.036 [−0.081, 0.009]	0.023	−1.565	.118
Parameters of interest: group comparisons using moderation models				
M_{HC1}: No language	0.016 [−0.133, 0.165]	0.076	0.205	.838
M_{HC1}: Language (vs. no language)	0.031 [−0.177, 0.239]	0.106	0.293	.770
M_{HC1}: No exercise	−0.027 [−0.180, 0.126]	0.079	−0.344	.731
M_{HC1}: Exercise (vs. no exercise)	0.111 [−0.110, 0.332]	0.113	0.986	.324
$M_{\Delta HC}$: No language	−0.053 [−0.108, 0.002]	0.027	−1.911	.056
$M_{\Delta HC}$: Language (vs. no language)	0.038 [−0.052, 0.128]	0.046	0.832	.405
$M_{\Delta HC}$: No exercise	−0.023 [−0.086, 0.040]	0.033	−0.706	.480
$M_{\Delta HC}$: Exercise (vs. no exercise)	−0.026 [−0.112, 0.060]	0.044	−0.588	.557

Note. Statistically significant parameters discussed in the text are bolded. Parameter names correspond to path labels in Figure 5a of the main text.

Supplementary Table S8 | *Model parameters: Latent change score model of cornu ammonis 3/dentate gyrus volume.*

Parameters: across all groups	Unstandardized estimate (*b*) [95% CI]	Standard error	*Z*-score	*p*-value
RES_{left1}	0.456 [0.330, 0.582]	0.064	7.125	.000
RES_{right1}	0.370 [0.280, 0.460]	0.046	8.043	.000
RES_{left2}	0.325 [0.257, 0.393]	0.035	9.286	.000
RES_{right2}	0.336 [0.251, 0.421]	0.044	7.636	.000
RES_{left3}	0.412 [0.287, 0.537]	0.064	6.437	.000
RES_{right3}	0.564 [0.434, 0.694]	0.066	8.545	.000
COV_{hemi}	0.230 [0.161, 0.299]	0.035	6.571	.000
VAR_{HC1}	0.607 [0.443, 0.771]	0.084	7.226	.000
$VAR_{\Delta HC}$	0.154 [0.094, 0.214]	0.031	4.968	.000
$COV_{HC1\sim\Delta HC12}$	−0.057 [−0.136, 0.022]	0.040	−1.425	.154
$COV_{HC1\sim\Delta HC23}$	−0.036 [−0.106, 0.034]	0.036	−1.000	.317
$COV_{\Delta HC12\sim\Delta HC23}$	−0.103 [−0.156, −0.050]	0.027	−3.815	.000
M_{HC1}	0.037 [−0.079, 0.153]	0.059	0.627	.531
$M_{\Delta HC}$ (Main effect of time)	−0.032 [−0.072, 0.008]	0.020	−1.600	.110
Parameters of interest: group comparisons using moderation models				
M_{HC1}: No language	−0.041 [−0.208, 0.126]	0.094	−0.476	.634
M_{HC1}: Language (vs. no language)	0.164 [−0.059, 0.387]	0.113	1.436	.151
M_{HC1}: No exercise	−0.064 [−0.223, 0.095]	0.081	−0.787	.431
M_{HC1}: Exercise (vs. no exercise)	0.197 [−0.052, 0.446]	0.127	1.558	.119
**$M_{\Delta HC}$: No language**	**−0.068 [−0.109, −0.027]**	**0.021**	**−3.183**	**.001**
**$M_{\Delta HC}$: Language (vs. no language)**	**0.082 [0.006, 0.158]**	**0.039**	**2.110**	**.035**
$M_{\Delta HC}$: Language only	0.014 [−0.088, 0.116]	0.052	0.270	.787
$M_{\Delta HC}$: Combined (vs. language only)	−0.008 [−0.149, 0.133]	0.072	−0.116	.908
$M_{\Delta HC}$: No exercise	−0.032 [−0.095, 0.031]	0.033	−1.006	.314
$M_{\Delta HC}$: Exercise (vs. no exercise)	0.000 [−0.080, 0.080]	0.041	0.012	.990

Note. Statistically significant parameters discussed in the text are bolded. Parameter names correspond to path labels in Figure 5a of the main text.

Supplementary Table S9 | *Model parameters: Latent change score model of subiculum volume.*

Parameters: across all groups	Unstandardized estimate (*b*) [95% CI]	Standard error	*Z*-score	*p*-value
RES_{left}	0.409 [0.339, 0.479]	0.036	11.361	.000
RES_{right}	0.409 [0.356, 0.462]	0.027	15.148	.000
COV_{hemi}	0.224 [0.169, 0.279]	0.028	8.000	.000
VAR_{HC1}	0.513 [0.369, 0.657]	0.074	6.932	.000
$VAR_{\Delta HC}$	0.115 [0.058, 0.172]	0.029	3.966	.000
$COV_{HC1\sim\Delta HC12}$	−0.003 [−0.077, 0.071]	0.038	−0.079	.937
$COV_{HC1\sim\Delta HC23}$	−0.031 [−0.095, 0.033]	0.032	−0.969	.333
$COV_{\Delta HC12\sim\Delta HC23}$	−0.051 [−0.098, −0.004]	0.024	−2.125	.034
M_{HC1}	0.017 [−0.088, 0.122]	0.053	0.321	.748
$M_{\Delta HC}$ (Main effect of time)	−0.031 [−0.069, 0.007]	0.020	−1.550	.121
**Parameters of interest: group comparisons using moderation models**				
M_{HC1}: No language	0.053 [−0.108, 0.214]	0.082	0.648	.517
M_{HC1}: Language (vs. no language)	−0.075 [−0.281, 0.131]	0.105	−0.720	.472
M_{HC1}: No exercise	−0.103 [−0.266, 0.060]	0.084	−1.236	.216
**M_{HC1}: Exercise (vs. no exercise)**	**0.234 [0.024, 0.444]**	**0.107**	**2.185**	**.029**
$M_{\Delta HC}$: No language	−0.059 [−0.102, −0.016]	0.022	−2.671	.008
$M_{\Delta HC}$: Language (vs. no language)	0.063 [−0.015, 0.141]	0.040	1.569	.117
$M_{\Delta HC}$: No exercise	−0.032 [−0.089, 0.025]	0.031	−1.120	.263
$M_{\Delta HC}$: Exercise (vs. no exercise)	0.002 [−0.076, 0.080]	0.039	0.061	.951

Note. Statistically significant parameters discussed in the text are bolded. Parameter names correspond to path labels in Figure 5a of the main text.

Supplementary Table S10 | Model parameters: Latent change score model of entorhinal cortex volume.

Parameters: across all groups	Unstandardized estimate (*b*) [95% CI]	Standard error	*Z*-score	*p*-value
RES_{left}	0.498 [0.411, 0.585]	0.045	11.067	.000
RES_{right}	0.550 [0.463, 0.637]	0.044	12.500	.000
COV_{hemi}	0.414 [0.330, 0.498]	0.043	9.628	.000
VAR_{HC1}	0.489 [0.361, 0.617]	0.065	7.523	.000
$VAR_{\Delta HC}$	0.049 [0.020, 0.078]	0.015	3.267	.001
$COV_{HC1\sim\Delta HC12}$	−0.090 [−0.138, −0.042]	0.024	−3.750	.000
$COV_{HC1\sim\Delta HC23}$	0.069 [0.023, 0.115]	0.024	2.875	.004
$COV_{\Delta HC12\sim\Delta HC23}$	−0.007 [−0.036, 0.022]	0.015	−0.467	.640
M_{HC1}	0.024 [−0.072, 0.120]	0.049	0.490	.624
$M_{\Delta HC}$ (Main effect of time)	−0.017 [−0.047, 0.013]	0.015	−1.133	.257
**Parameters of interest: group comparisons using moderation models**				
M_{HC1}: No language	0.083 [−0.066, 0.232]	0.078	1.088	.277
M_{HC1}: Language (vs. no language)	−0.126 [−0.349, 0.097]	0.129	−1.111	.267
M_{HC1}: No exercise	−0.093 [−0.232, 0.046]	0.076	−1.310	.190
**M_{HC1}: Exercise (vs. no exercise)**	**0.227 [0.027, 0.427]**	**0.102**	**2.222**	**.026**
$M_{\Delta HC}$: No language	−0.015 [−0.056, 0.026]	0.020	−0.712	.476
$M_{\Delta HC}$: Language (vs. no language)	−0.005 [−0.066, 0.056]	0.031	−0.161	.872
$M_{\Delta HC}$: No exercise	−0.046 [−0.099, 0.007]	0.027	−1.717	.086
$M_{\Delta HC}$: Exercise (vs. no exercise)	0.055 [−0.010, 0.120]	0.033	1.686	.092

Note. Statistically significant parameters discussed in the text are bolded. Parameter names correspond to path labels in Figure 5a of the main text.

Supplementary Table S11 | *Model parameters: Latent change score model of Mnemonic Discrimination Task for Objects and Scenes performance.*

Parameters: across all groups	Unstandardized estimate (*b*) [95% CI]	Standard error	*Z*-score	*p*-value
$LOAD_2$	0.9162 [0.775, 1.149]	0.095	10.126	.000
$LOAD_3$	1.018 [0.853, 1.183]	0.084	12.119	.000
RES	0.602 [0.556, 0.648]	0.024	25.083	.000
VAR_{MDT1}	0.497 [0.359, 0.635]	0.071	7.000	.000
$VAR_{\Delta MDT}$	0.131 [0.032, 0.230]	0.051	2.569	.010
$COV_{MDT1\sim\Delta MDT12}$	−0.150 [−0.226, −0.074]	0.039	−3.846	.000
$COV_{MDT1\sim\Delta MDT23}$	−0.024 [−0.097, 0.049]	0.037	−0.649	.516
$COV_{\Delta MDT12\sim\Delta MDT23}$	−0.034 [−0.120, 0.052]	0.044	−0.773	.440
M_{MDT1}	−0.079 [−0.180, 0.022]	0.052	−1.519	.129
**$M_{\Delta MDT}$ (Main effect of time)**	**0.068 [0.015, 0.121]**	**0.027**	**2.519**	**.012**

Parameters of interest: group comparisons using moderation models				
M_{MDT1}: No language	−0.086 [−0.221, 0.049]	0.069	−1.255	.209
M_{MDT1}: Language (vs. no language)	0.015 [−0.156, 0.186]	0.087	0.173	.863
M_{MDT1}: No exercise	−0.114 [−0.249, 0.021]	0.067	−1.642	.101
M_{MDT1}: Exercise (vs. no exercise)	0.069 [−0.096, 0.234]	0.084	0.827	.408
$M_{\Delta MDT}$: No language	0.066 [0.001, 0.131]	0.033	1.990	.047
$M_{\Delta MDT}$: Language (vs. no language)	0.005 [−0.085, 0.095]	0.046	0.116	.908
$M_{\Delta MDT}$: No exercise	0.022 [−0.047, 0.091]	0.035	0.621	.535
**$M_{\Delta MDT}$: Exercise (vs. no exercise)**	**0.089 [0.005, 0.173]**	**0.043**	**2.070**	**.038**
$M_{\Delta MDT}$: Exercise only	0.081 [−0.007, 0.169]	0.045	1.794	.073
$M_{\Delta MDT}$: Combined (vs. exercise only)	0.024 [−0.094, 0.142]	0.061	0.401	.688

Note. Statistically significant parameters discussed in the text are bolded. Parameter names correspond to path labels in Figure 5b of the main text.

Supplementary Table S12 | Model parameters of interest: bivariate associations between hippocampal subfield volume and Mnemonic Discrimination Task for Objects and Scenes performance.

Model	Parameter: across all groups	Standardized estimate (*β* or *ρ*)	Standard error	*Z*-score	*p*-value
CA1/CA2 volume ~ MDT-OS	$COV_{HC1{\sim}MDT1}$	0.089 [−0.083, 0.261]	0.088	1.011	.312
	$REG_{MDT1 \rightarrow HC\Delta}$	0.068 [−0.075, 0.211]	0.072	0.932	.351
	$REG_{HC1 \rightarrow MDT\Delta}$	0.090 [−0.151, 0.331]	0.121	0.732	.464
	$COV_{\Delta HC \rightarrow \Delta MDT}$	−0.190 [−0.594, 0.214]	0.206	−0.922	.357
CA3/DG volume ~ MDT-OS	$COV_{HC1{\sim}MDT1}$	0.136 [−0.017, 0.289]	0.078	1.744	.081
	$REG_{MDT1 \rightarrow HC\Delta}$	0.024 [−0.074, 0.122]	0.049	0.480	.631
	$REG_{HC1 \rightarrow MDT\Delta}$	0.054 [−0.164, 0.272]	0.111	0.486	.627
	$COV_{\Delta HC \rightarrow \Delta MDT}$	−0.142 [−0.403, 0.119]	0.136	−1.068	.286
Subiculum volume ~ MDT-OS	$COV_{HC1{\sim}MDT1}$	0.001 [−0.179, 0.181]	0.091	0.011	.991
	$REG_{MDT1 \rightarrow HC\Delta}$	0.075 [−0.050, 0.200]	0.063	1.172	.241
	$\mathbf{REG_{HC1 \rightarrow MDT\Delta}}$	**0.291 [0.044, 0.538]**	**0.107**	**2.310**	**.021**
	$COV_{\Delta HC \rightarrow \Delta MDT}$	−0.189 [−0.522, 0.144]	0.170	−1.112	.266
ERC volume ~ MDT-OS	$COV_{HC1{\sim}MDT1}$	−0.041 [−0.253, 0.171]	0.112	−0.380	.704
	$REG_{MDT1 \rightarrow HC\Delta}$	0.046 [−0.140, 0.232]	0.094	0.484	.628
	$REG_{HC1 \rightarrow MDT\Delta}$	0.210 [−0.037, 0.457]	0.126	1.667	.096
	$COV_{\Delta HC \rightarrow \Delta MDT}$	0.150 [−0.132, 0.432]	0.144	1.042	.297

Model	Parameters compared across groups using multi-group models (not bootstrapped)				
Subiculum volume ~ MDT-OS	$REG_{HC1 \rightarrow MDT\Delta}$: No exercise	0.353 [0.065, 0.641]	0.144	2.401	.016
	$REG_{HC1 \rightarrow MDT\Delta}$: Exercise	0.230 [-0.058, 0.518]	0.148	1.565	.118

Note. CA = cornu ammonis, DG = dentate gyrus, ERC = entorhinal cortex, MDT(-OS) = Mnemonic Discrimination Task (for Objects and Scenes), COV = covariance path, REG = regression path, HC = model-specific hippocampal subfield.
Statistically significant parameters discussed in the text are bolded. Parameter names correspond to path labels in Figure 6 of the main text.

Supplementary Figures

Supplementary Figure S1 | Univariate (boxplot) and multivariate (spaghetti plot) outliers (blue). Univariate outliers were defined as those falling > 4 SDs away from the mean. Multivariate outliers were determined using the classical product-moment method (criterion = .001).

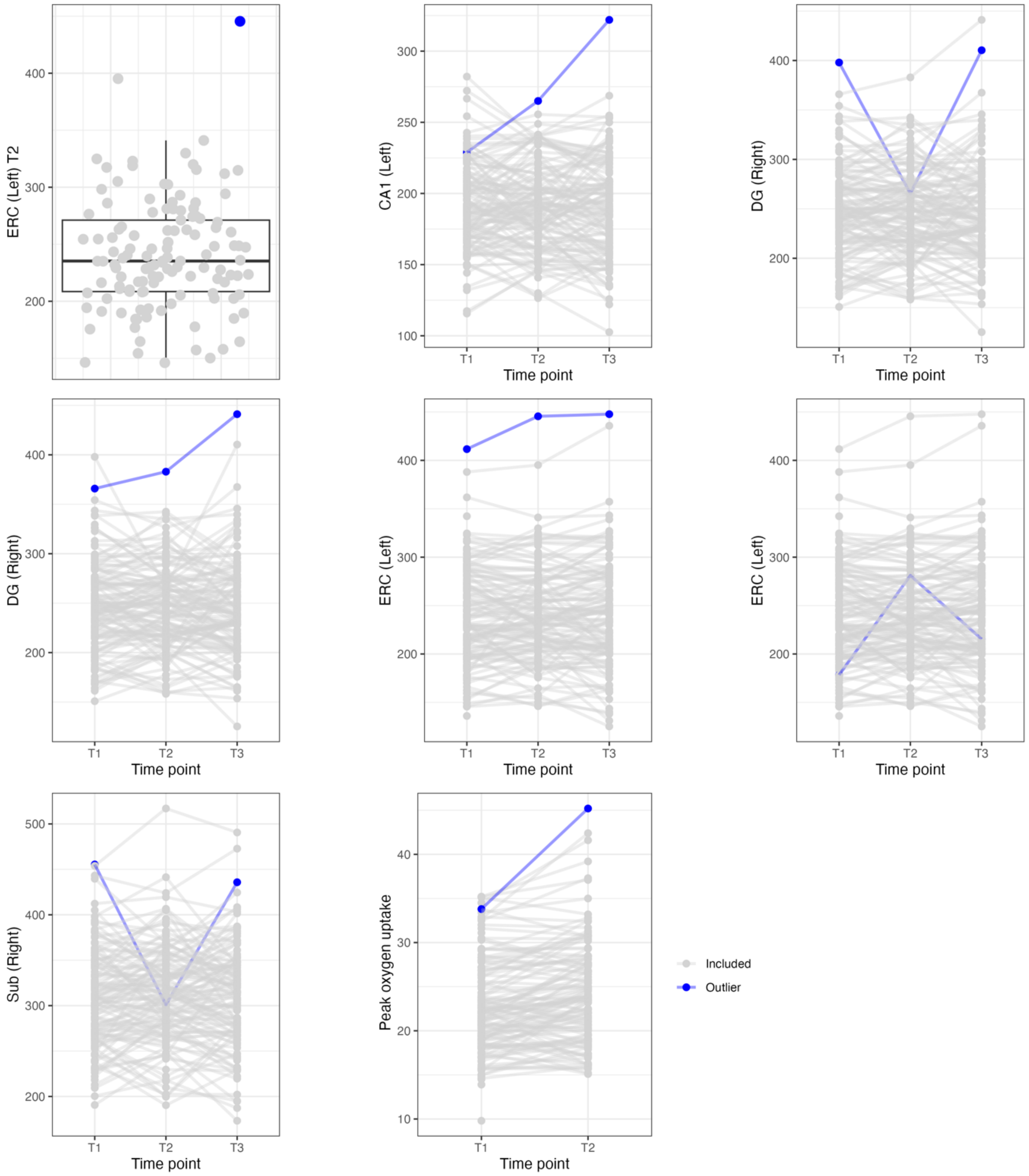

Supplementary Figure S2 | Distribution of manifest variables at each time point (violin) and individual change trajectories (spaghetti), as well as the mean (black dot) and standard deviation (error bars) at each time point and for each group.

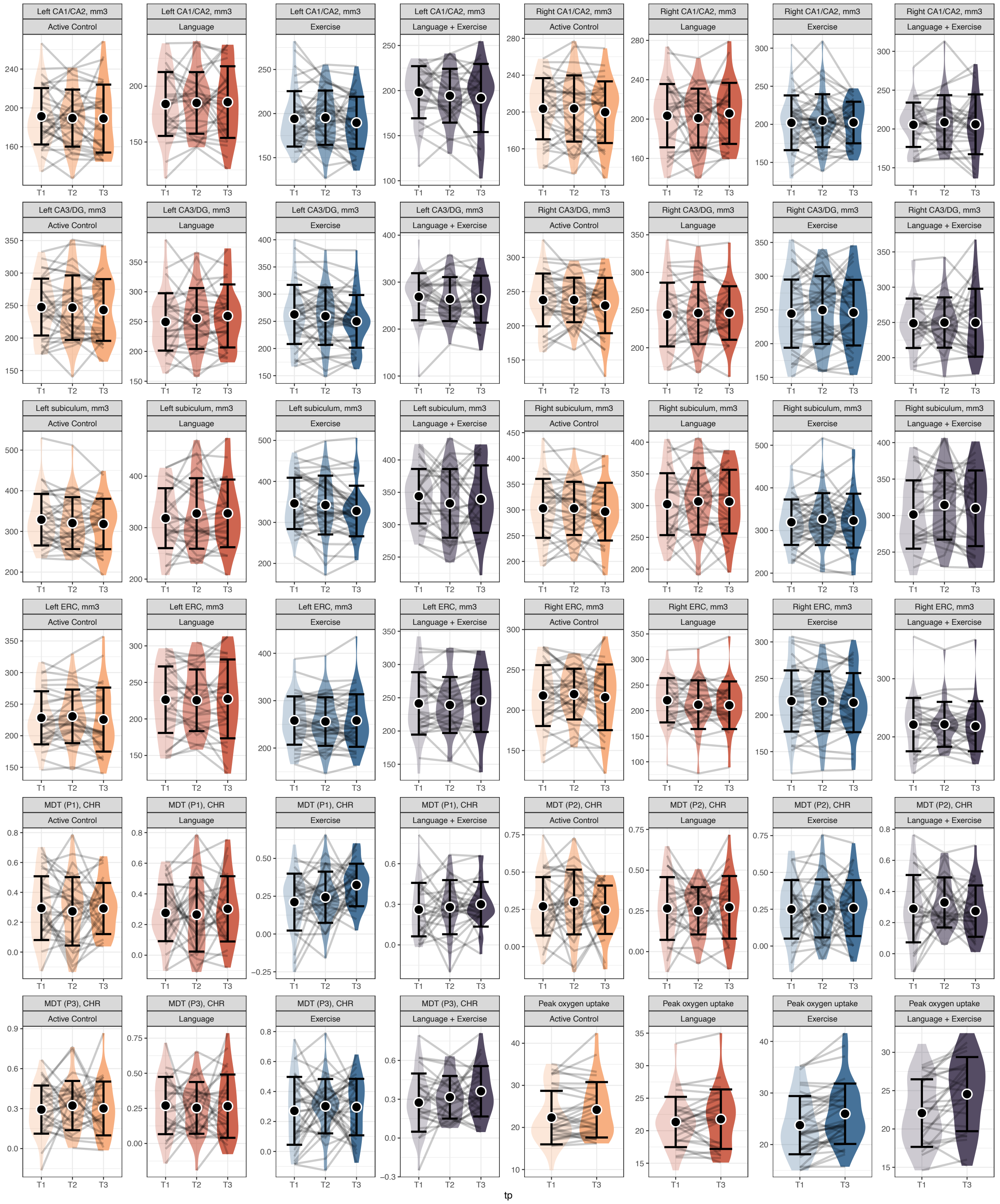

Note. CA = *cornu ammonis*, DG = dentate gyrus, ERC = entorhinal cortex, MDT = Mnemonic Discrimination Task, P = parcel, CHR = corrected hit rate.

Supplementary Figure S3 | Bivariate latent change score models (LCSMs) including (a) covariates of no interest (age, sex, years of education) or (b) change in cardiovascular fitness as a pseudo-LCSM.

(a)

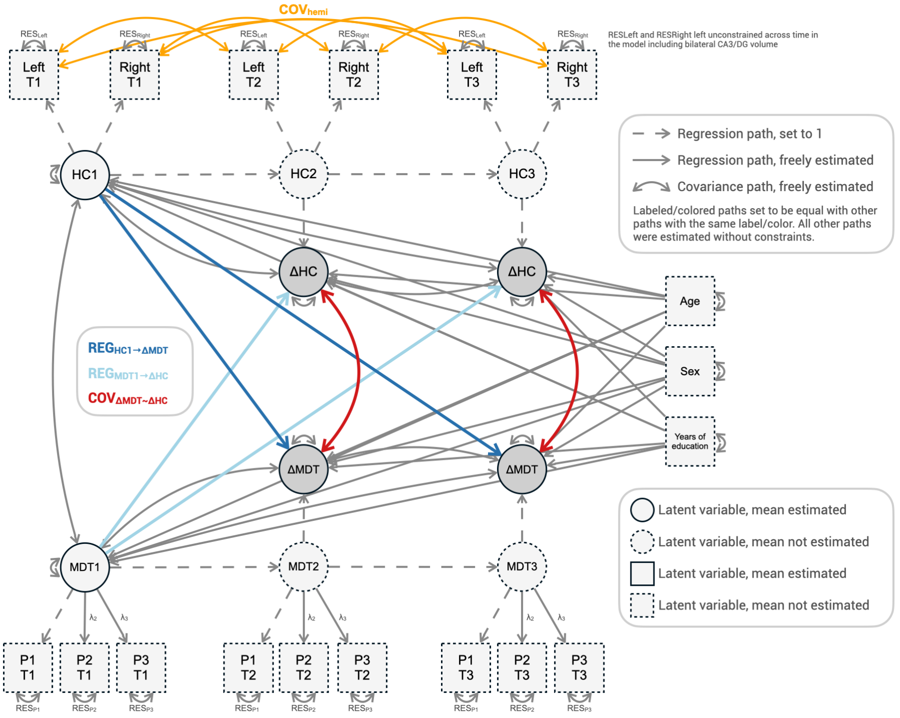

(b)

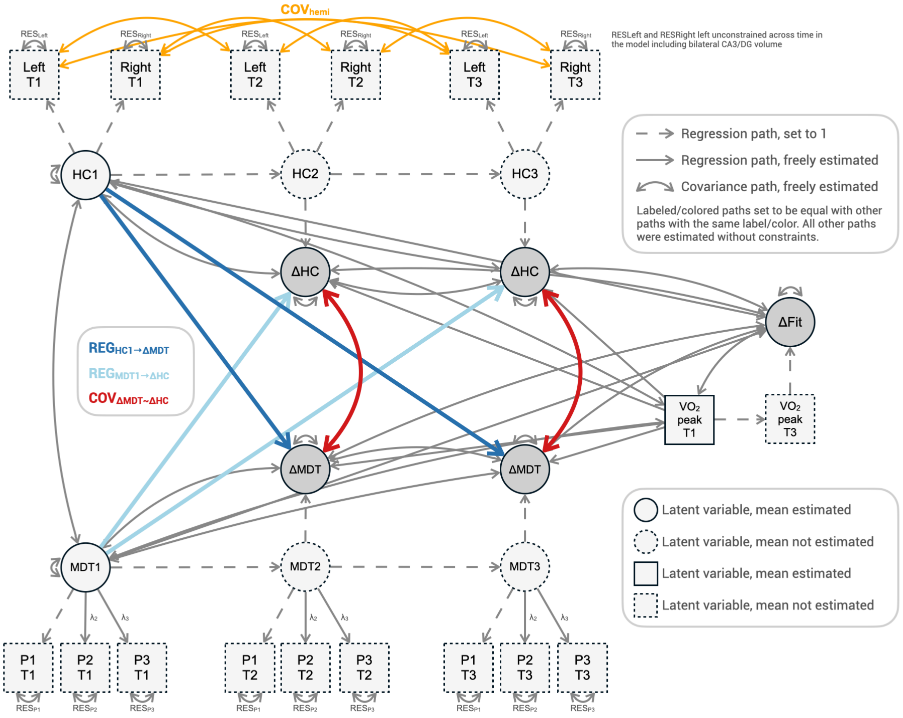

Note. HC = hippocampus, MDT = Mnemonic Discrimination Task, P = parcel, COV = covariance, REG = regression, RES = residual, λ = loading. Models were run separately for *cornu ammonis* 1/2, *cornu ammonis* 3/dentate gyrus, subiculum, and entorhinal cortex.

Supplementary Methods

Measuring physical fitness

Physical fitness was quantified as peak oxygen uptake (VO_2peak), relativized by body weight in kilograms (Dickhuth & Badtke, 2010), which is a standard measurement of endurance performance (Bassett & Howley, 2000). To assess VO_2peak, ergospirometry was performed using the Quark Cardio Pulmonary Exercise Test (CPET; COSMED) with the standard Breath-by-Breath setup and V2 Mask (Hans Rudolph, Inc.), using OMNIA software (Version 1.6.2, COSMED), while participants pedaled at a constant rate of 60–70 RPM on a bicycle ergometer for a three-minute rest phase, followed by an exertion phase with a starting resistance of 20 W, which increased by 20 W every three minutes until participants reported reaching maximum exertion, and a final five-minute recovery phase with no resistance (Bosquet et al., 2002; Braumann et al., 2004).

Measuring baseline cognitive function

The Digit Symbol Substitution Task (DSST; Wechsler, 1981) was administered at all three timepoints. Here, we used the DSST as a general measure of cognitive health (Jaeger, 2018) to ensure that all intervention groups had comparable cognitive function at baseline. Participants were presented with a code box with nine digit-symbol pairs and rows of digits with corresponding blank boxes under each digit. They were instructed to draw the associated symbols below each digit without skipping any boxes as quickly as possible within 90 seconds. One point was awarded for each correctly drawn symbol, and participants were penalized one point for each incorrectly drawn symbol.

Imaging data acquisition and preprocessing

Brain images were acquired using a 3T Magnetom Trio MRI scanner system (Siemens Medical Systems, Erlangen, Germany; software version VB17a) using a 32-channel radiofrequency (RF) head coil. Identical sequences were acquired at all three time points.

A T1-weighted 3D magnetization-prepared rapid gradient echo (MPRAGE) sequence was acquired parallel to the genu-splenium axis of the corpus callosum on the sagittal plane, with the following parameters: TR = 2,500 ms, TE = 4.77 ms, inversion time (TI) = 1,100 ms, flip angle = 7°, isotropic voxel size = 1.0 mm^3, using unfiltered images and pre-scan normalization, matrix size = 192 × 256 × 256, no parallel imaging, 7/8 partial Fourier acquisition, bandwidth = 140 Hz/pixel, and TA = 9:20 min.

A high-resolution, T2-weighted 2D turbo spin echo (TSE) sequence localized on the hippocampus was acquired, oriented perpendicularly to the long axis of the right hippocampus, with the following parameters: in-plane resolution = 0.4 × 0.4 mm, slice thickness = 2.0 mm, 31 coronal slices, image matrix 384 × 384, repetition time (TR) = 8,510 ms, echo time (TE) = 50 ms, flip

angle = 120°, turbo factor = 15 applying hyperechoes, bandwidth = 99 Hz/pixel, 1 average per acquisition, and acquisition time (TA) = 7:13 min.

The anteroposterior extent of the bilateral hippocampal bodies was determined separately in each image by two raters who were blinded to participant ID, training group, and measurement time point. The anterior limit was defined as the first slice posterior to the uncal apex on which neither the uncus nor tissue of the hippocampal head was visible and no partial volume artifacts could be identified and the posterior limit was defined as the furthest posterior slice on which the lamina quadrigemina was visible (Bender et al., 2018). Raters trained on random subsets of data until they showed substantial agreement across hemispheres and for both anterior and posterior limits (κs = .633–.874, ps < .001) before evaluating all hippocampal images. ASHS segmentation was applied using a customized atlas that has previously been validated for use in samples of older adults (Bender et al., 2018). This segmentation protocol, although implemented without taking within-subject measurements into account, has shown excellent test-retest reliability and a high sensitivity to detect change after two years in a sample of children (Homayouni et al., 2021). To correct for segmentation errors at the anterior and posterior limits of the hippocampal body, data were truncated to only those slices within the manually defined ranges, as this has been found to substantially improve the validity of automated segmentations (Bender et al., 2018).

Right and left CA1/CA2, CA3/DG, subiculum, and ERC volumes in mm^3 were then extracted (see Figure 3 of the main text). A random selection of segmentations was visually inspected and found to be satisfactory. Finally, volumes of each subfield were adjusted for intracranial volume (ICV, calculated using the *Estimate TIV and global tissue volumes* module in CAT12) using an analysis of covariance approach (Raz et al., 2005): adjusted volume = raw volume − b × (ICV − mean ICV), where b is the slope of regression of subfield volume on ICV. Adjusted subfield volumes were used in all analyses.

Measuring mnemonic discrimination

To assess mnemonic discrimination, the Mnemonic Discrimination Task for Objects and Scenes (MDT-OS; adapted from Berron et al., 2018) was administered at all three time points. Stimuli (everyday indoor objects or empty scenes; computer-generated with 3ds Max, Autodesk Inc., San Rafael, USA; isoluminant) were presented to participants sequentially to test participants' ability to discriminate between identical and similar lure stimuli. A single trial comprised the presentation of four stimuli (object or scene): (1) a first novel stimulus, (2) a second novel stimulus, (3) either the first stimulus or a lure resembling the first stimulus, (4) either the second stimulus or a lure resembling the second stimulus. Stimuli were presented on a touchscreen for 3,000 ms with jittered interstimulus intervals (ISI) of 400 to 1,600 ms (mean ISI = 999 ms) between each presentation. While the third and

fourth stimuli were being presented, participants were asked to indicate with a touchscreen stylus whether the stimulus was identical to the previously shown stimulus (repeat) or not (lure). If they thought the image was a repeat stimulus, they were asked to press an “Identical” button displayed below the image. If they thought the image was a lure stimulus, they were asked to indicate where in the image the stimulus was different from the one previously presented. The exact coordinates of where participants indicated were not considered when evaluating whether a lure object was correctly rejected or not; it was sufficient if participants recognized that the stimulus had changed. Object stimuli could differ in shape, but not color, position, or size, and scene stimuli could differ in geometry, but not color or viewpoint. Participants completed 56 trials over two blocks for a total of 224 stimuli (56 object first presentations, 28 object repeat, 28 object lure, 56 scene first presentations, 28 scene repeat, 28 scene lure), counterbalanced and randomized across object/scene and repeat/lure. Identical stimuli were presented at all three time points.

Multivariate latent change score models

Bilateral volume at T1, T2, and T3 was captured as a latent variable loading onto volume in the right and left hemispheres of the respective subfield, with both loadings fixed to 1. Intercepts of the manifest variables were not explicitly modeled, as the observed data were centered around 0. Additionally, the residual covariance between measurements at T1, T2, and T3 was estimated, with coefficients constrained to be equal. Residual variance was allowed to differ between hemispheres but was assumed to be equal across time points for those models with strict factorial invariance longitudinally. Variance parameters were estimated with a lower bound of .00001. Before analysis, to ensure factorial invariance across groups and time points, invariance tests were conducted using a sequence of likelihood-ratio tests (LRT; Cheung & Rensvold, 1999; Widaman et al., 2010). To conduct a multi-group LRT, a model in which the parameter of interest is freely estimated within each group is compared to a nested model in which this parameter is set to be equal across groups. The difference in model fit (i.e., the log-likelihood ratio) between the two models, if significant, indicates that the null hypothesis that the models fit equally well can be rejected (Kline, 2016). First, the factor structures at T1 were tested for strict factorial invariance across the four groups (i.e., identical residual variances across groups; note that factor loadings were set to 1 in the bilateral hippocampal subfield volume models so testing for metric factorial invariance was not necessary). Then, groups were collapsed and the longitudinal factor structure was tested for strict factorial invariance across the three time points (i.e., identical residual variances across time points). To test for group differences in change, latent variables were used to capture the change from T1 to T2 and from T2 to T3. These latent variables were allowed to correlate with the observed score at T1, as well as with each other. Mean change in performance and variance in change was constrained to be equal between T1 to T2 and T2 to T3 after

using LRTs to test the assumption that change and variance in change would not differ between the two timeframes. The parameters for variance in change were tested against 0 using LRTs to ensure sufficient variance to test for associations with change.

Similar LCSMs were used to evaluate training-related differences in change in MDT-OS performance using the parceling approach described in the main text (Little et al., 2002, 2013), treating each parcel as a separate indicator variable. The difficulty of each MDT-OS item was determined using data from Güsten et al. (2021, Cortex), in which a large sample across a wide age range (N = 1554, age range = 18–77 years) performed an online version of the MDT-OS. The hit rates in this separate sample were used to estimate the difficulty of each item, with higher hit rates corresponding to easier items. Items were then binned into easy, medium, and hard items, with an equal number of items in each bin. Parcels were then assigned equal numbers of easy, medium, and hard items. Item assignment to parcels was identical at all time points. In this way, the overall parcel difficulty was kept consistent across parcels and time points. At T1, T2, and T3 a latent variable predicting each of the three parcels was built to capture MDT-OS performance on a latent level, parsing out measurement error. The loading from latent MDT-OS score to corrected hit rate in one of the parcels, arbitrarily labeled parcel 1, was fixed to 1 and the loadings to the other two parcels were freely estimated, constrained to be equal across time points. Before analysis, we tested for metric (i.e., identical factor loadings) and strict factorial invariance (i.e., identical factor loadings and residual variances) across groups and time points using LRTs (Cheung & Rensvold, 1999). Additionally, residual variance was constrained to be equal across parcels after testing and confirming this assumption. The same structural model as described above was used to test for group differences at baseline and in change in mnemonic discrimination performance.

Supplementary Results

Regarding the interpretation of model estimates

Parameter estimates can be interpreted as such: In the case of main effects of time, unstandardized estimates (*b*) can be interpreted as the extent to which the dependent variable changes for every time unit increase (e.g., in the current analyses, $b = .200$ indicates that every three months, the independent variable increases by 20% of an SD). In the case of group comparisons, as the data were standardized to have a mean of 0 and a standard deviation (SD) of 1, *b* can be interpreted as the difference in change in the independent variable across the groups (e.g., $b = .200$ indicates that the test group changes 20% of an SD more than the reference group for every 1 SD change in the independent variable). Standardized estimates of covariance between variables (ρ) can be interpreted in the same way as a correlation coefficient. In the case of regression paths between latent baseline values and change, standardized estimates of regression (*β*) as the degree to which the dependent variable changes for every SD change in the independent variable (e.g., $\beta = .200$ indicates that the dependent variable changes 20% of an SD for every 1 SD change in the independent variable compared to the mean). Estimates are statistically significant if the 95% confidence interval does not include zero.

Regarding baseline differences in hippocampal subfield volume.

We found that exercisers (EG and L+EG) showed greater volume in the bilateral subiculum, b = 0.234, 95% CI [0.024, 0.444], and ERC, b = 0.227, 95% CI [0.027, 0.427] than non-exercisers (ACG and LG). This difference was not explained by sex, despite the great percentage of females in the language group; volumes were adjusted for intracranial volume and did not show any systematic differences across males and females (*t*s = −1.68 to 0.81, *p*s > .096 across subfields and time points). As the groups were assigned randomly (with the exception of couples and friends who were assigned to the same groups because of the blinding), we can only speculate that this difference is also random and would be attenuated in larger groups.

Sensitivity analyses: outliers and compliance

A number of outliers were removed before running the current analyses: one data point was removed following univariate outlier detection (> 4 SDs away from the mean) and six cases of subfield volume data and one case of VO_2peak data were removed (i.e., all time points from the outlying individual). Sensitivity analyses including these data points were conducted, and results remained largely unchanged. The CA3/DG effect of language was found to be marginal ($p = .068$) but in the expected direction, with language learners showing maintenance of CA3/DG volume. The MDT-OS effect of exercise remained significant ($p = .039$), with exercisers showing a greater increase in mnemonic discrimination performance. The predictive relationship between baseline subiculum

volume and change in MDT-OS performance, irrespective of training type, was also preserved (*p* = .023). No other parameters were found to be significant.

Another set of sensitivity analyses were conducted, fully excluding those individuals who did not pass the compliance threshold (*n* = 16; > 1,890 minutes of study-related activity). Results were again unchanged by excluding these individuals. The effect of language learning on CA3/DG volume was found to be significant (*p* = .046), as was the effect of exercise on MDT-OS performance (*p* = .041). Finally, the predictive relationship found between baseline subiculum volume and change in MDT-OS performance was again preserved (*p* = .013). Again, no additional parameters were found to be significant.

Brain-derived neurotrophic factor does not predict mnemonic discrimination

Serum brain-derived neurotrophic factor (BDNF) was collected from the current sample at T1 and T3 (see Wenger et al., 2022 for details), however we found no evidence that baseline levels of or change in serum BDNF was associated with MDT-OS. No training-specific changes (group-by-time interaction tested with ANOVA with age, sex, and years of education as covariates) were observed, $F_{(3, 124)} = 1.23$, $p = .302$. A set of post-hoc analyses was run using models identical to the analysis including VO_2peak as a pseudo-latent LSCM in the multivariate LCSM (see main text for details). All models showed acceptable fit, RMSEAs ≤ .028, CFIs ≥ .985. However, neither serum BDNF levels at T1 nor change in serum BDNF was significantly associated with baseline levels or changes in MDT-OS performance. Including BDNF in the model did not account for the relationship between subiculum volume at T1 and change in Item-Context performance, which remained significant, $\beta = 0.298$, 95% CI [0.043, 0.553].

Supplementary References

Bassett, D. R., & Howley, E. T. (2000). Limiting factors for maximum oxygen uptake and determinants of endurance performance: Medicine & Science in Sports & Exercise, 70. https://doi.org/10.1097/00005768-200001000-00012

Bender, A. R., Keresztes, A., Bodammer, N. C., Shing, Y. L., Werkle-Bergner, M., Daugherty, A. M., Yu, Q., Kühn, S., Lindenberger, U., & Raz, N. (2018). Optimization and validation of automated hippocampal subfield segmentation across the lifespan. Human Brain Mapping, 39(2), 916–931. https://doi.org/10.1002/hbm.23891

Berron, D., Neumann, K., Maass, A., Schütze, H., Fliessbach, K., Kiven, V., Jessen, F., Sauvage, M., Kumaran, D., & Düzel, E. (2018). Age-related functional changes in domain-specific medial temporal lobe pathways. Neurobiology of Aging, 65, 86–97. https://doi.org/10.1016/j.neurobiolaging.2017.12.030

Bosquet, L., Léger, L., & Legros, P. (2002). Methods to Determine Aerobic Endurance: Sports Medicine, 32(11), 675–700. https://doi.org/10.2165/00007256-200232110-00002

Braumann, K.-M., Ziegler, M., & Reer, R. (2004). FREIZEIT- UND FITNESSSPORT. Sports Orthopaedics and Traumatology Sport-Orthopädie - Sport-Traumatologie, 20(2), 71–75. https://doi.org/10.1078/0949-328X-00199

Cheung, G. W., & Rensvold, R. B. (1999). Testing Factorial Invariance across Groups: A Reconceptualization and Proposed New Method. Journal of Management, 25(1), 1–27. https://doi.org/10.1177/014920639902500101

Dickhuth, H.-H., & Badtke, G. (Eds.). (2010). Sportmedizin für Ärzte: Lehrbuch auf der Grundlage des Weiterbildungssystems der Deutschen Gesellschaft für Sportmedizin und Prävention (DGSP) (2nd ed.). Deutscher Ärzteverlag.

Homayouni, R., Yu, Q., Ramesh, S., Tang, L., Daugherty, A. M., & Ofen, N. (2021). Test–retest reliability of hippocampal subfield volumes in a developmental sample: Implications for longitudinal developmental studies. Journal of Neuroscience Research, 99(10), 2327–2339. https://doi.org/10.1002/jnr.24831

Jaeger, J. (2018). Digit Symbol Substitution Test: The Case for Sensitivity Over Specificity in Neuropsychological Testing. Journal of Clinical Psychopharmacology, 38(5), 513–519. https://doi.org/10.1097/JCP.0000000000000941

Kline, R. B. (2016). Principles and practice of structural equation modeling (4th ed.). The Guilford Press.

Little, T. D., Cunningham, W. A., Shahar, G., & Widaman, K. F. (2002). To Parcel or Not to Parcel: Exploring the Question, Weighing the Merits. Structural Equation Modeling: A Multidisciplinary Journal, 9(2), 151–173. https://doi.org/10.1207/S15328007SEM0902_1

Little, T. D., Rhemtulla, M., Gibson, K., & Schoemann, A. M. (2013). Why the items versus parcels controversy needn't be one. Psychological Methods, 18(3), 285–300. https://doi.org/10.1037/a0033266

Raz, N., Lindenberger, U., Rodrigue, K. M., Kennedy, K. M., Head, D., Williamson, A., Dahle, C., Gerstorf, D., & Acker, J. D. (2005). Regional Brain Changes in Aging Healthy Adults: General

Trends, Individual Differences and Modifiers. Cerebral Cortex, 15(11), 1676–1689. https://doi.org/10.1093/cercor/bhi044

Schulz, K. F., Altman, D. G., Moher, D., & for the CONSORT Group. (2010). CONSORT 2010 Statement: Updated guidelines for reporting parallel group randomised trials. BMJ, 340(mar23 1), c332–c332. https://doi.org/10.1136/bmj.c332

Wechsler, D. (1981). The psychometric tradition: Developing the Wechsler adult intelligence scale. Contemporary Educational Psychology, 6(2), 82–85. https://doi.org/10.1016/0361-476X(81)90035-7

Wenger, E., Düzel, S., Polk, S. E., Bodammer, N. C., Misgeld, C., Porst, J., Wolfarth, B., Kühn, S., & Lindenberger, U. (2022). Vamos en bici: Study protocol of an investigation of cognitive and neural changes following language training, physical exercise training, or a combination of both [Preprint]. bioRxiv. https://doi.org/10.1101/2022.01.30.478181

Widaman, K. F., Ferrer, E., & Conger, R. D. (2010). Factorial Invariance Within Longitudinal Structural Equation Models: Measuring the Same Construct Across Time. Child Development Perspectives, 4(1), 10–18. https://doi.org/10.1111/j.1750-8606.2009.00110.x
